# An 8-step procedure-specific risk framework enables reproducible biosafety level assignment beyond agent-based classification

**DOI:** 10.3389/fbioe.2026.1879247

**Published:** 2026-07-17

**Authors:** Rafael Cena-Diez

**Affiliations:** 1 Centre for Infectious Medicine, Department of Medicine Huddinge, Karolinska Institutet, Stockholm, Sweden; 2 Faculty of Medicine, University of Valladolid, Valladolid, Spain

**Keywords:** biological risk assessment, biosafety, biosafety level assignment, laboratory risk management, laboratory safety, procedure-specific risk, risk-based containment

## Abstract

**Objective:**

Current biosafety frameworks that directly link Risk Group (RG) to Biosafety Level (BSL) fail to capture how much exposure varies across the procedures performed in modern biomedical laboratories operating with genetically modified organisms, viral vectors, and multi-step protocols. This article presents the Procedure-Specific Risk (PSR) framework, an 8-step operational protocol for reproducible Biosafety Level assignment in which the exposure generated by the procedure—rather than agent taxonomy—serves as the primary determinant of containment.

**Methods:**

A structured comparative analysis of ten national and international biosafety reference documents was conducted (WHO Laboratory Biosafety Manual 4th ed., BMBL 6th ed., CDC Biological Risk Assessment 2024, INSST Technical Guide 2024, and relevant EU and Spanish legislation). Conceptual convergence was evaluated through qualitative thematic synthesis. The resulting 8-step protocol integrates agent Risk Group classification, procedural exposure characterization, and modulating factor evaluation into a BSL assignment matrix, and is supported by a structured assessment template and a freely accessible bilingual digital implementation tool.

**Validation:**

Framework validation rests on three complementary forms of evidence: content validity (all components derived from the ten analyzed regulatory sources), convergent validity (PSR-derived BSL assignments consistent with WHO and BMBL recommendations across all six case studies), and coverage validity (purposive case selection spanning RG1–3, Low–High PSR, escalation and reduction scenarios, and dual-technology comparison). Prospective multi-institutional inter-rater reliability assessment (target κ ≥ 0.60) constitutes the planned next validation step, supported by the digital implementation tool.

**Conclusion:**

The PSR framework provides a structured, reproducible, and immediately applicable protocol for proportionate containment in bioengineering and biotechnology settings. It is compatible with existing institutional biosafety programs and requires no structural regulatory modification for adoption. Implementation is supported by a freely accessible bilingual web tool, a structured assessment template, and six worked examples covering RG1–3 agents and diverse procedural risk levels.

## 1 Introduction

Biological risk assessment in research and clinical laboratories has traditionally relied on agent-based classification: the inherent danger of a microorganism, its pathogenicity, transmissibility, and available treatments, determines the required containment level. Under this model, each pathogen is assigned to a Risk Group (RG1–4), and that Risk Group directly dictates the required Biosafety Level (BSL): a Risk Group 2 organism automatically calls for BSL-2 containment, regardless of how it is handled. The field is increasingly moving away from this one-to-one mapping, recognizing that it fails to capture the complex interplay of pathogen hazard, technical operations, and operational context. Traditional methods that directly link Risk Group (RG) to Biosafety Level (BSL) requirements present well-documented limitations in modern laboratories, which employ diverse protocols, genetically modified organisms (GMOs), and viral vector systems (Caskey and Sevilla-Reyes, 2015; European and Council of the European U, 2009; Kimman et al., 2008; National Research, 1989; Trump, 2017).

European Union Directives 2000/54/EC and 2009/41/EC, incorporated into Spanish law through RD 664/1997 and RD 178/2004, reinforce the necessity for procedure-specific evaluation and proportionate risk management (European and Council of the European U, 2009; España, 1997; España, 2004; European et al., 2000; Instituto Nacional de Seguridad y Salud en el T, 2024a). The Procedure-Specific Risk (PSR) framework (known as ‘Riesgo Específico del Procedimiento (REP)' in Spain),where PSR is defined as the level of biological exposure risk generated by a specific laboratory procedure, determined independently of the agent’s taxonomic classification, addresses an important gap in practice (Centers for Disease C et al., 2020; Instituto Nacional de Seguridad y Salud en el T, 2022; Instituto Nacional de Seguridad y Salud en el T, 2024; Instituto Nacional de Seguridad y Salud en el T, 2024b; World Health, 2020). Although international agencies endorse this approach, the literature lacks a consolidated conceptual synthesis and standardized implementation methods. Furthermore, the terminology used for this concept is inconsistent across regulatory documents: the WHO Laboratory Biosafety Manual (2020) uses “risk assessment by procedure”, the BMBL 6th edition refers to “procedure-specific risk”, and the CDC Biological Risk Assessment framework (2024) employs “likelihood evaluation by procedure” (see Table 4). This terminological heterogeneity impedes international harmonization and reproducible BSL assignment. At the international governance level, the Cartagena Protocol on Biosafety (Association for the Advancement of Medical I, 2016) provides an overarching framework for the safe handling of living modified organisms, reinforcing the need for structured risk evaluation methodologies that transcend agent-based classification (Centers for Disease C et al., 2020; World Health, 2020; Association for the Advancement of Medical I, 2016; Centers for Disease C and Prevention, 2024; Secretariat of the Convention on, 2000; Select Agents, 2025).

This article advances a structured operational framework developed through a systematic comparative analysis of ten key national and international reference documents, including the WHO Laboratory Biosafety Manual 4th ed. (2020), the CDC/NIH Biosafety in Microbiological and Biomedical Laboratories (BMBL) 6th ed. (2020), the CDC Biological Risk Assessment (2024), the INSST Technical Guide (2024), and relevant EU Directives and Spanish legislation (Instituto Nacional de Seguridad y Salud en el T, 2024a; Centers for Disease C et al., 2020; Instituto Nacional de Seguridad y Salud en el T, 2024c; World Health, 2020; Association for the Advancement of Medical I, 2016; Centers for Disease C and Prevention, 2024; Select Agents, 2025). By formalizing procedure-driven exposure as a quantifiable determinant of containment, this work contributes to an emerging decision science for laboratory biosafety governance through an 8-step structured methodology intended to support defensible and reproducible BSL assignment (Instituto Nacional de Seguridad y Salud en el T, 2024a; Centers for Disease C et al., 2020; World Health, 2020; Centers for Disease C and Prevention, 2024). The framework introduces BSL-2+ as an operationally defined intermediate containment tier, formally described in Section 4.4. To our knowledge, no prior work has presented a consolidated stepwise operational framework that systematically translates international regulatory convergence on procedure-specific risk into a reproducible decision model for BSL assignment. Existing guidelines endorse the underlying principles but leave their operational integration to institutional discretion; the PSR framework bridges that gap.

### 1.1 Conceptual evolution and limitations of agent-based models

Historical biosafety frameworks established in the mid-20th century prioritized biological agent classification as the primary determinant of containment (National Institutes of, 2019; World Health, 1983). However, several limitations of purely agent-based systems have become apparent:

Novel Risk Profiles: Genetic modifications, such as third-generation replication-defective lentiviral vectors, introduce risk profiles that are not captured by parental-organism classification (Bouard et al., 2009; Kotterman et al., 2015; Naldini, 1998; Sinn et al., 2005; Wang et al., 2019).

Exposure Variability: Identical agents manipulated through different procedures present vastly different exposure probabilities, such as Mycobacterium tuberculosis in sealed cultures versus sonication of concentrated pellets (Pottage et al., 2022; Ravnholdt et al., 2025; Wurtz et al., 2016).

Operational Factors: Variables including volume, concentration, aerosol potential, and operator competency alter actual risk independently of agent taxonomy (Pottage et al., 2022; Ravnholdt et al., 2025; Wurtz et al., 2016).

Inefficiency: Rigid RG–BSL correlations often result in over-containment and resource waste or, conversely, under-protection and personnel exposure (Kimman et al., 2008; National Research, 1989; Cameron et al., 2014; Pike, 1976).

**FIGURE 1:**
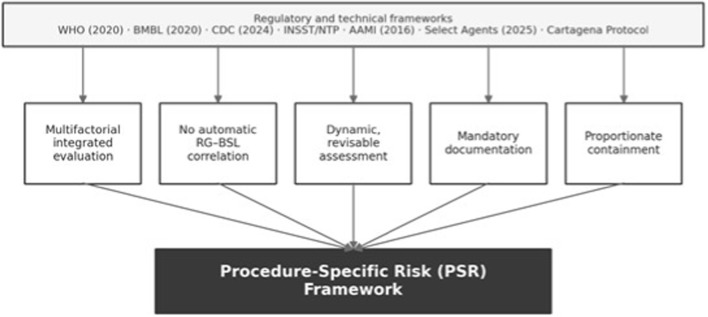
Conceptual convergence underpinning the PSR framework. Ten national and international biosafety reference documents (WHO, BMBL, CDC, INSST, and relevant EU and Spanish legislation) converge on five core principles (Caskey and Sevilla-Reyes, 2015): multifactorial risk integration (European and Council of the European U, 2009); rejection of automatic RG-to-BSL correlation (Kimman et al., 2008); dynamic revisability upon procedural changes (National Research, 1989); mandatory written documentation; and (Trump, 2017) proportionate containment. These principles collectively ground the 8-step PSR framework. (BSL, Biosafety Level; RG, Risk Group; PSR, Procedure-Specific Risk).

The PSR framework addresses these misalignments by making procedural exposure, rather than taxonomy, the primary determinant of containment, translating the risk-proportionate logic of modern regulations into a structured decision model (Instituto Nacional de Seguridad y Salud en el T, 2024a; Centers for Disease C et al., 2020; World Health, 2020; Centers for Disease C and Prevention, 2024; International Organization for S, 2019). This shift is exemplified by the evolution of the WHO Laboratory Biosafety Manual: the 3rd edition (2004) (World Health, 2004) retained a largely prescriptive scheme anchored in agent Risk Group, whereas the 4th edition (2020) formalized an evidence- and risk-based approach that explicitly decouples BSL from automatic RG correlation and centers assessment on the activity performed. Binding legislation reflects the same logic: European Directive 2000/54/EC (transposed in Spain by Royal Decree 664/1997) flags certain Group 3 agents with two asterisks (**) to indicate that they may present only a limited risk of infection because they are not normally infectious by the airborne route, an explicit recognition that route of transmission, not Risk Group alone, governs the containment actually required. The PSR framework proposed here operationalizes this transition into a standardized stepwise model. Analysis of regulatory frameworks revealed explicit convergence across international and national sources on five fundamental principles. Figure 1 illustrates how five shared principles—multifactorial risk integration, rejection of automatic RG-to-BSL correlation, dynamic revisability, mandatory documentation, and proportionate containment—collectively underpin the PSR framework across all ten analyzed sources (European and Council of the European U, 2009; España, 1997; España, 2004; European et al., 2000; Instituto Nacional de Seguridad y Salud en el T, 2024a; Centers for Disease C et al., 2020; Instituto Nacional de Seguridad y Salud en el T, 2024c; World Health, 2020; Centers for Disease C and Prevention, 2024). Supplementary Material includes practical examples of completed risk assessments. Risk-based biosafety is widely practiced, but decisions often remain organism-focused, creating uncertainty in biosafety level selection particularly for procedures whose risk profiles diverge from organism-based assumptions. The present framework directly addresses this misalignment by providing a standardized, stepwise protocol that operationalizes procedure-specific risk evaluation for institutional use.

## 2 Materials and equipment

### 2.1 Regulatory and technical reference documents

The following documents are required to implement the PSR framework and must be consulted during Steps 1–6 of the protocol. All are publicly available at no cost:

- WHO Laboratory Biosafety Manual, 4th ed. (World Health Organization, 2020)
- Biosafety in Microbiological and Biomedical Laboratories (BMBL), 6th ed. (CDC/NIH, 2020)
- Biological Risk Assessment Process, Safe Labs Portal (CDC, 2024)
- Guía técnica para la evaluación y prevención de los riesgos relacionados con la exposición a agentes biológicos, 3^a^ ed. (INSST, 2024)
- NTP 1201: Riesgo biológico: utilización confinada de organismos modificados genéticamente (INSST, 2024)
- Real Decreto 664/1997 sobre la protección de los trabajadores contra los riesgos relacionados con la exposición a agentes biológicos durante el trabajo (España, 1997)
- Directive 2000/54/EC on the protection of workers from risks related to exposure to biological agents at work (European Parliament and Council, 2000)
- Directive 2009/41/EC on the contained use of genetically modified micro-organisms (European Parliament and Council, 2009)

### 2.2 Biological agent classification databases

Risk Group classification of biological agents (Step 1) must be verified against at least one authoritative source. Recommended databases include the WHO risk group classifications (Annex 2, WHO Laboratory Biosafety Manual 4th ed.), the BMBL agent summary statements (Supplementary Appendix B), and the INSST biological agents database (https://www.insst.es/agentes-biologicos).

### 2.3 Assessment template

The structured PSR assessment template provided in Supplementary Appendix S1 is required to complete Steps 1–8 of the protocol. The template is available as an editable document and must be completed in full before submission to the Institutional Biosafety Committee (IBC). Successful implementation additionally requires: (a) access to the biological agent’s technical data sheet or equivalent documentation, (b) detailed written description of all laboratory procedures to be assessed (standard operating procedures or equivalent), and (c) IBC authorization prior to initiating work at any assigned BSL.

### 2.4 Digital implementation tool

A freely accessible bilingual (EN/ES) web-based implementation tool is available at: https://rafael-67.github.io/psr-tool/. The tool guides the user sequentially through the 8-step PSR decision logic, applies the classification matrices in Tables 6–8, and generates a downloadable structured assessment record for institutional use. Multi-user mode enables simultaneous independent assessments of the same scenario and computes inter-rater agreement (Cohen’s kappa and Fleiss’ kappa) in real time, with κ ≥ 0.60 (substantial agreement) as the operational validation threshold. No installation is required; the tool operates entirely client-side with no data retention. The tool is a decision-support instrument intended for biosafety professionals and Institutional Biosafety Committees (IBCs) to document and standardize expert assessment; it is not a lay self-assessment application. Use of the digital tool is recommended but not mandatory: the structured assessment template (Supplementary Appendix S1) may be used independently for paper-based or offline implementation.

### 2.5 Cross-source alignment summary

Supplementary Appendix S4 provides a structured tabular summary of cross-source alignment for the five fundamental PSR principles across all ten analyzed regulatory documents. This table supports reproducibility of the convergence analysis described in Section 3.1.3 and may be used as a reference checklist when adapting the framework to additional jurisdictional contexts.

### 2.6 Additional jurisdictional resources

Users operating outside European and North American jurisdictions should additionally consult applicable national frameworks. Relevant examples include China’s Biosecurity Law (2020) and associated laboratory biosafety standards (GB 19489-2008), and Japan’s biosafety provisions under the Cartagena Act (2003, revised 2019). While these documents were not part of the formal ten-source analytical corpus of this study, their underlying risk-based principles are consistent with the five PSR convergence principles identified in Section 3.1.3, and they may serve as jurisdictionally appropriate complements to the core reference documents listed above.

## 3 Methods

### 3.1 Objective and scope

The PSR framework provides a standardized 8-step protocol for biosafety level assignment in diagnostic and research laboratory settings. The primary objective is to replace automatic Risk Group (RG)–to–BSL correlation with a structured, procedure-specific exposure assessment that produces documented, defensible, and reproducible containment decisions. The protocol is applicable to natural biological agents (RG1–4), genetically modified organisms, and viral vector systems. It does not cover industrial-scale production, animal biosafety levels (ABSL), fieldwork, or biosecurity threat assessment.

### 3.2 Regulatory basis, document selection, and convergence analysis

This work constitutes a structured regulatory synthesis rather than a formal systematic review. The selection methodology described below was designed to ensure representativeness across regulatory jurisdictions and methodological traditions, but does not claim compliance with PRISMA or equivalent systematic review protocols. The analytical approach follows established methods for integrative framework analysis in regulatory science, including comparative regulatory mapping and qualitative thematic synthesis (Arksey and O'Malley, 2005; Greenhalgh et al., 2018). Methodological scope and limitations are detailed in Sections 6.3, 6.4. The analysis is intentionally delimited to European and North American (here understood as the United States and Canada; Latin American jurisdictions, while geographically part of the Americas, were outside the scope of this initial synthesis and are identified as a priority for subsequent comparative work) regulatory frameworks (WHO, US CDC/NIH, EU Directives, Spanish INSST) on the grounds that these jurisdictions have produced the most methodologically developed procedure-specific biosafety assessment frameworks to date. Asia-Pacific regulatory frameworks were identified as a priority for inclusion in subsequent comparative analyses but fell outside the jurisdictional scope defined for this initial synthesis. Databases consulted: PubMed, Scopus, Web of Science, the INSST repository, the WHO institutional library, and the CDC Safe Labs portal. Search period: January 1997 to December 2025. Search terms: (“procedure-specific risk” OR “procedure-specific risk assessment” OR “risk assessment by procedure”) AND (“biosafety” OR “biosafety level” OR “BSL assignment”) AND (“laboratory” OR “microbiological”). This yielded 46 records for screening.

#### 3.2.1 Selection criteria

Inclusion criteria: Documents were included if they: (a) provided regulatory or technical guidance on biological risk assessment; (b) addressed biosafety level assignment methodology; (c) discussed the relationship between agent classification and procedural risk; (d) originated from governmental agencies, international organizations, or recognized professional bodies; (e) were published in English or Spanish.

Exclusion criteria: Documents were excluded if they: (a) focused exclusively on biosecurity without biosafety components; (b) addressed only environmental or agricultural biosafety; (c) lacked a clear methodological framework; (d) were opinion articles; (e) consisted solely of organism-specific fact sheets without risk assessment methodology.

Of the 14 documents meeting eligibility criteria, four were excluded during final corpus delimitation: two due to conceptual redundancy with already-included sources from the same regulatory tradition, and two due to scope mismatch. Final corpus composition was determined prior to data extraction (Tables 1, 2). Table 1 documents the document-selection process (identification, screening, and eligibility), ensuring the transparency and reproducibility of the regulatory synthesis. The regulatory architecture underpinning the PSR framework—spanning international governance, supranational law, national implementation, and technical guidance—is summarized in Table 3.

**TABLE 1:** Document selection summary.

Stage	Records	Excluded	Included
Identification	46	22	24
Screening	24	10	14
Eligibility	14	4	10

**TABLE 2:** Comparative regulatory frameworks supporting Procedure-Specific Risk (PSR) assessment.

Source	Scope	Procedure focus	Agent–procedure–BSL relationship	Dynamic review	Contribution to PSR	Jurisdiction
RD 664/1997 (Spain)	Legal	Implicit	Evaluation by activity	Yes	Provides a legal basis	Spain
INSST guide 2024	National technical	Explicit	Integrated BSL assignment	Yes	Primary methodological reference	Spain
NTP 1201 (INSST)	National technical	Explicit (GMOs)	Formal matrix	Yes	Operationalization	Spain
WHO 2020	International	Explicit	Risk-based BSL	Yes	Provides a conceptual foundation	International
BMBL 2020	International	Explicit	Formal combination	Yes	Serves as a reference model	United States of America
CDC 2024	International	Highly explicit	Quantitative likelihood	Yes	Probability basis	United States of America
NTP 875 (INSST)	National technical	Implicit	Risk evaluation by equipment and procedure	Yes	Methodological complement for equipment-based risk	Spain
NTP 1202 (INSST)	National technical	Explicit	Equipment-procedure integration	Yes	Safety equipment selection by procedure type	Spain
AAMI 2016	International	Explicit	Integrated risk assessment	Yes	Professional consensus on procedural hazards	International
Select agents 2025 (CDC/USDA)	International	Explicit	Procedure-based hazard evaluation	Yes	Regulatory model for high-consequence agents	United States of America

**TABLE 3:** Regulatory architecture underpinning Procedure-Specific Risk (PSR) assessment.

Level	Framework/Source	Regulatory nature	Core risk logic	Role in PSR
International governance	Cartagena protocol on biosafety; WHO laboratory biosafety manual	Binding treaty	Macro-level biosafety oversight	Establishes global biorisk governance
Supranational law	Directives 2000/54/EC and 2009/41/EC	EU legislation	Mandatory risk evaluation and proportional containment	Provides the legal foundation for risk-based biosafety
National implementation	Royal decrees 664/1997 and 178/2004 (Spain)	Statutory law	Activity- and procedure-based assessment	Operationalizes biological risk within domestic regulation
Organizational standard	ISO 35001:2019	Certifiable management system	Institutional biorisk governance	Embeds PSR within auditable organizational structures
Conceptual frameworks	WHO laboratory biosafety manual (2020); BMBL (2020)	Authoritative guidelines	Integrated agent–procedure evaluation	Supply the theoretical and operational backbone of PSR
Emerging analytical models	CDC biological risk assessment (2024)	Technical guidance	Probabilistic risk modeling (likelihood × severity)	Supports structured and defensible containment decisions
National technical adaptation	INSST technical guide (2024); NTP 1201	Technical guidance	Matrix-driven procedural assessment	Enables practical implementation at the laboratory level

#### 3.2.2 Data extraction and synthesis

Conceptual convergence was operationally defined as explicit or implicit endorsement of procedure-based biological risk evaluation within the framework of biosafety level determination. Variables extracted included procedural emphasis, BSL assignment methodology, documentation requirements, and dynamic reassessment criteria. A structured qualitative thematic synthesis was performed. Thematic convergence analysis was performed by the corresponding author, with conceptual validation through discussion with co-authors.

#### 3.2.3 International convergence on PSR principles

Across the ten references analyzed, we observed consistent alignment on five fundamental PSR principles (see Supplementary Appendix S4 for a structured summary of cross-source alignment; Table 2 in the main article for the comparative regulatory overview): (a) multifactorial integration (Risk = f[Agent, Procedure, Context]); (b) rejection of automatic RG–BSL correlation; (c) dynamic revisability upon procedural changes; (d) mandatory written documentation; and (e) proportionate containment matching operational risk. All analyzed sources—WHO, BMBL, INSST, CDC, and AAMI—endorsed these principles either explicitly or implicitly. This convergence across jurisdictions and institutional traditions reinforces the conceptual robustness of procedure-specific risk evaluation.

Beyond the ten formally analyzed sources, the same procedure-based logic is reflected in additional national systems, supporting the international scope of these principles. Canada’s Canadian Biosafety Standard and Canadian Biosafety Handbook (PHAC), together with PHAC Pathogen Safety Data Sheets, embed activity- and procedure-based assessment. In Asia, China’s Biosecurity Law (2020) and Japan’s biosafety provisions under the Cartagena Act (2003, rev. 2019) likewise adopt risk-based principles. National transposition of supranational requirements also varies: while EU Directives 2000/54/EC and 2009/41/EC set minimum standards, Belgium operates a unified biosafety framework covering both pathogens and GMOs, Spain implements them through Royal Decrees 664/1997 and 178/2004 with INSST technical guidance, and Sweden through the Swedish Work Environment Authority provisions. In practice, these transpositions differ meaningfully: Spain’s dual-decree structure is complemented by detailed INSST technical guidance documents (including classification matrices and worked examples) that operationalize procedure-specific risk at the bench level, whereas Sweden’s implementation through Arbetsmiljöverket follows a more principles-based occupational health framework without an equivalent national technical guidance document of comparable methodological specificity.

In the United States, by contrast, laboratory biosafety is governed predominantly at the federal level (BMBL, NIH Guidelines, Federal Select Agent Program), so inter-state variation is comparatively limited. These differences in administrative implementation do not alter the underlying convergence on procedure-based risk evaluation.

#### 3.2.4 Terminological variations across jurisdictions

While conceptual convergence is evident, terminology varies significantly across sources (Table 4). The term “Procedure-Specific Risk (PSR)” or its Spanish equivalent “Riesgo Específico del Procedimiento (REP)” represents a structured operational synthesis absent from international documents, which use dispersed formulations: “risk assessment by procedure” (WHO), “procedure-specific risk” (BMBL), “likelihood evaluation by procedure” (CDC), and “procedural hazards” (Select Agents Program). This terminological heterogeneity impedes standardization and underscores the need for international harmonization (International Organization for S, 2019).

**TABLE 4:** Terminological variations in Procedure-Specific Risk assessment.

Source	Term used	Equivalence to PSR
WHO (2020)	“Risk assessment by procedure”	Conceptually aligned with PSR
BMBL (2020)	“Procedure-specific risk”	Conceptually aligned with PSR
CDC (2024)	“Likelihood evaluation by procedure”	Operational component of PSR
AAMI (2016)	“Hazards from procedure”	Conceptually aligned with PSR
Select agents (2025)	“Procedure hazards”	Operational component of PSR
INSST (2024)	“Riesgo específico del procedimiento”	Operational component of PSR (original term)

“Conceptually aligned” indicates that the source uses distinct but equivalent terminology for the same construct. “Operational component” indicates that the source explicitly operationalizes one dimension of PSR (likelihood estimation or hazard evaluation) without employing a unified PSR, construct; RD, 664/1997 and EU, Directives 2000/54/EC, and 2009/41/EC, provide the legal mandate for procedure-based evaluation but do not employ specific PSR, terminology and are therefore not listed in this table; their role is described in Table 3.

### 3.3 Protocol: 8-Step PSR framework for BSL assignment

**FIGURE 2:**
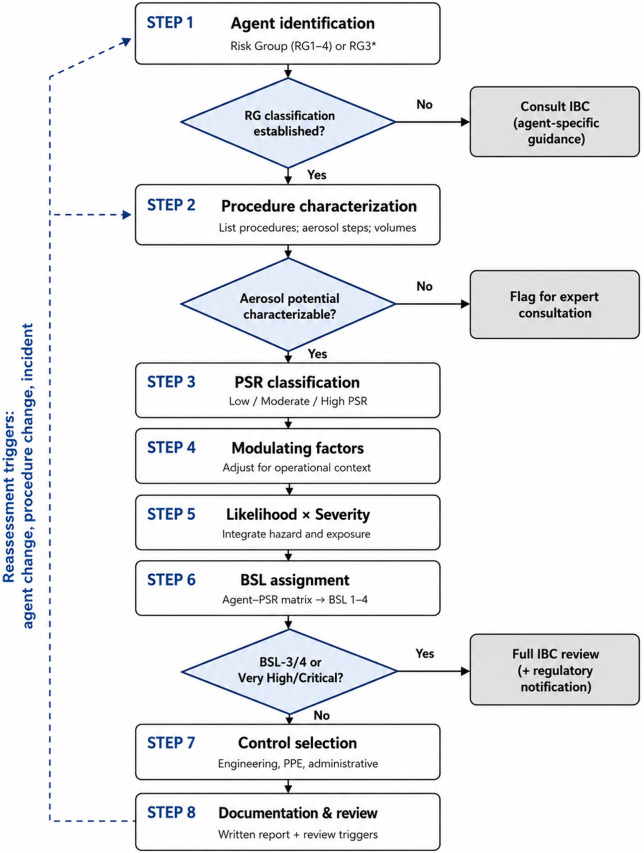
Eight-step PSR assessment framework for BSL assignment. Sequential flow diagram from agent identification (Step 1) through documentation and review (Step 8). Diamond nodes indicate three mandatory decision points (pause points at Steps 1, 2, and 6). Dashed lines represent reassessment triggers that reactivate the protocol from Step 1 or Step 2. (BSL, Biosafety Level; RG, Risk Group; PSR, Procedure-Specific Risk; IBC, Institutional Biosafety Committee; PPE, Personal Protective Equipment).

The PSR framework was developed to address a fundamental limitation of agent-based classification: that the same organism can generate vastly different exposure probabilities depending on how it is manipulated, and that containment decisions should therefore be driven by procedural exposure likelihood rather than taxonomy alone. The protocol must be completed in sequential order for each biological procedure or set of procedures under assessment. A structured overview of all eight steps is provided in Table 5. Use the structured assessment template (Supplementary Appendix S1) to document each step. All completed assessments require review and approval by the Institutional Biosafety Committee before laboratory work begins. The framework presumes an Institutional Biosafety Committee (IBC), or the equivalent national or institutional authority, responsible for reviewing and authorizing the written risk assessment, particularly for BSL-2+ assignments and any escalation or de-escalation decision. Total estimated time for a complete assessment is 3–6 h depending on protocol complexity; individual step estimates are provided as planning guidance. Throughout this framework, ‘PSR level’ (Low, Moderate, or High) denotes the procedural exposure category assigned to each manipulation, distinct from the ‘Biosafety Level’ (BSL-1 through BSL-4), which represents the final containment assignment derived by integrating the agent Risk Group with the PSR level. The framework operationalizes the fundamental equation Risk = Hazard × Likelihood of Exposure, where hazard is determined by agent classification and likelihood is explicitly operationalized through PSR classification (Figure 2). Three mandatory pause points interrupt the sequential flow to require institutional consultation before proceeding: absence of an established RG classification (Step 1), uncharacterizable aerosol generation potential (Step 2), and BSL-3/4 output or Very High/Critical overall risk level (Step 6) (España, 1997; European et al., 2000; Instituto Nacional de Seguridad y Salud en el T, 2024a; Centers for Disease C et al., 2020; Instituto Nacional de Seguridad y Salud en el T, 2024c; World Health, 2020; Centers for Disease C and Prevention, 2024; Select Agents, 2025).

**TABLE 5:** Structured overview of the 8-step PSR operational framework for biosafety level assignment.

Step	Purpose	Key input	Output
1. Agent identification	Classify a biological hazard	Agent data, RG databases	Risk group (RG1–4) or RG3*
2. Procedure characterization	Map exposure scenarios	Laboratory SOPs	Procedure list with parameters
3. PSR classification	Estimate exposure likelihood	Aerosol, volume, concentration	Low/Moderate/High PSR
4. Modulating factors	Adjust for operational context	9 procedural modulators	PSR level confirmed or escalation triggered (BSL upgrade considered)
5. Likelihood × severity	Integrate hazard and exposure	RG + PSR level (with escalation if applicable)	Overall risk level
6. BSL assignment	Determine containment	Agent–PSR matrix (Table 8)	BSL-1 through BSL-4
7. Control selection	Specify protective measures	BSL requirements	Engineering, PPE, and admin controls
8. Documentation	Ensure traceability	Assessment record	Written report + review triggers

#### 3.3.1 Step 1 — agent identification (estimated time: 30–60 min)

Determine the Risk Group (RG1–4) of the biological agent using at least one authoritative source (WHO, BMBL, or INSST database). For genetically modified organisms, evaluate the parental organism classification alongside the nature of genetic modification, replication competence, and environmental stability, applying the dominant containment principle. Document the classification source explicitly. Assign RG3* to RG3-derived agents with validated attenuation (replication incompetence, confirmed absence of airborne transmissibility, or validated inactivation) where applicable (conditions governing BSL-2+ assignment to RG3* agents are detailed in Step 6).

Pause point: If the agent lacks an established RG classification (e.g., novel engineered organism, gain-of-function variant), do not proceed. Consult the IBC for agent-specific guidance before continuing.

#### 3.3.2 Step 2 — procedure characterization (estimated time: 30–90 min)

List all technical procedures involved in the planned work. For each procedure, document: working volume (mL), pathogen concentration (CFU/mL, PFU/mL, TU/mL, or MOI as applicable), aerosol generation potential (minimal/moderate/high), equipment used, and procedural frequency. Identify the highest-risk procedural step—this step determines the maximum PSR level assigned in Step 3.

Pause point: If procedures involve steps whose aerosol generation potential cannot be characterized (e.g., novel microfluidic systems at scale), flag for expert consultation before PSR classification.

#### 3.3.3 Step 3 — PSR classification (estimated time: 15–30 min)

Assign a PSR level (Low, Moderate, or High) to each procedure using the standardized classification matrix (Table 6). Classification is based on three parameters: aerosol generation potential, working volume, and pathogen concentration. The highest PSR level assigned across all procedures in the assessment determines the overall PSR classification carried forward to Steps 4 and 5.

**TABLE 6:** Procedure classification matrix defining PSR exposure categories based on aerosol generation, volume, and concentration**.**

PSR level	Aerosol generation	Volume and concentration thresholds	Representative procedures
LOW	Minimal (controlled conditions)	<10 mL; <10^6^ CFU/PFU/mL	Slow pipetting in BSC; plate seeding in BSC; Sample dilution in BSC
MODERATE	Moderate (contained)	10–100 mL; 10^6^–10^8^ CFU/PFU/mL	Sealed centrifugation; moderate vortexing (closed tubes); cell transfection in BSC; low MOI viral infection
HIGH	High (uncontained)	>100 mL; >10^8^ CFU/PFU/mL	Unsealed centrifugation; sonication; intense vortexing; pressurized tube opening; high-titer virus concentration; high MOI infection

Threshold values represent synthesis of quantitative data from WHO (2020), BMBL (2020), CDC (2024), and documented institutional practices. Volume categories (<10 mL, 10–100 mL, >100 mL) reflect WHO, recommendations for small-scale vs. large-scale operations. Aerosol generation classifications integrate particle-generation studies (Sinn et al., 2005; Pottage et al., 2022) and the risk-proportionate framework outlined in CDC (2024). Concentration thresholds align with typical diagnostic, research, and production-scale titers. Proposed values require empirical validation through environmental monitoring and exposure assessment studies. Classification logic: PSR, level is determined by the highest-scoring parameter; a single parameter at High level is sufficient to classify the overall procedure as High PSR, regardless of whether other parameters fall in lower categories (see Case Study 4, where high aerosol generation during sonication overrides the moderate volume threshold).

#### 3.3.4 Step 4 — modulating factors evaluation (estimated time: 20–40 min)

Evaluate the nine procedural modulating factors listed in Table 7. Record whether each factor is present in a risk-increasing condition. Count the total number of unfavorable factors among the nine procedural modulators.

**TABLE 7:** Procedural modulating factors influencing exposure likelihood in PSR assessment.

Factor	Increases PSR	Decreases PSR
Volume	>100 mL	<10 mL
Concentration	>10^8^ CFU/PFU/mL	<10^6^ CFU/PFU/mL
Frequency	Daily/Weekly	Occasional
Aerosol generation	High (sonication, vortex)	Minimal (slow pipetting)
Environmental stability	High (AAV, spores)	Low (lentivirus)
Operator experience	<6 months	>2 years
Supervision	No direct supervision	Direct, continuous
Infrastructure	Inadequate BSL	Adequate infrastructure with appropriate primary containment equipment
Incident history	Previous exposures	No incidents

Decision rule: If ≥ 2 unfavorable procedural modulating factors are present, containment escalation must be considered (e.g., BSL-2 → BSL-2+, or BSL-2+ → BSL-3). Individual susceptibility (immunocompromised status, absence of available vaccination) is documented separately and does not count toward this threshold; it must be addressed through occupational health measures at Step 8.

#### 3.3.5 Step 5 — likelihood–hazard integration (estimated time: 15–30 min)

Combine the procedural exposure likelihood (derived from PSR level and modulating factors, Step 4) with the agent hazard (severity, derived from RG classification, Step 1) using the risk matrix in Supplementary Appendix S1, Section 6. Assign an overall risk level (Negligible, Low, Moderate, High, Very High, or Critical).

#### 3.3.6 Step 6 — BSL assignment (estimated time: 15–30 min)

Apply the Agent–PSR combination matrix (Table 8) to assign the biosafety level. BSL-2+ assignments require simultaneous fulfillment of all four conditions specified in Table 8 footnotes. For RG3 agents, BSL-3 is the default; BSL-2+ is permissible only under Low PSR with all four conditions met, and only for agents with documented attenuation (RG3*).

**TABLE 8:** Biosafety level assignment matrix (risk group × PSR**)**.

Risk group	Low PSR	Moderate PSR	High PSR
RG1	BSL-1	BSL-1*	BSL-2**
RG2	BSL-2	BSL-2	BSL-2+
RG3	BSL-2+***	BSL-3	BSL-3
RG3*	BSL-2+	BSL-2+/BSL-3	BSL-3
RG4	BSL-4	BSL-4	BSL-4

* RG1 + Moderate PSR: standard BSL-1, practices; enhanced documentation recommended when working at scale. ** RG1 + High PSR: BSL-2, practices apply exclusively to the high-aerosol-generating step; all other steps remain BSL-1. Requires documented procedure-specific risk assessment. *** RG3 + Low PSR: BSL-2+ is permissible ONLY, when ALL, four conditions are simultaneously met: (a) documented risk assessment with explicit written justification; (b) IBC, approval; (c) full implementation of enhanced controls (Class II BSC, mandatory, double gloves, eye protection, respiratory protection); and (d) documented procedure-specific operator competency. BSL-3, remains the DEFAULT, for all RG3 agents. RG3* denotes an RG3 agent with documented attenuation (replication incompetence, confirmed absence of airborne transmissibility, or validated inactivation). BSL-3, is mandatory for High PSR, regardless of attenuation status.

Pause point: If the matrix output is BSL-3 or BSL-4, or if the overall risk level from Step 5 is Very High or Critical, do not proceed without full IBC review and, where applicable, regulatory notification.

#### 3.3.7 Step 7 — control selection (estimated time: 30–60 min)

Specify all control measures required for the assigned BSL, organized into three categories: (a) engineering controls (biosafety cabinet class, room pressure, HEPA filtration, sealed centrifuge rotors, autoclave access); (b) personal protective equipment (glove type, eye protection, respiratory protection, gown type); (c) administrative controls (approved SOPs, documented training and competency assessment, access restrictions, medical surveillance program, incident reporting system, waste management plan, emergency response plan). Minimum requirements per containment tier are detailed in Table 9.

**TABLE 9:** Operational differentiation of BSL-2, BSL-2+, and BSL-3 containment tier**s**.

Feature	BSL-2	BSL-2+	BSL-3
Primary containment	BSC-II recommended	BSC-II mandatory for all procedures	BSC-II mandatory; class III for high-aerosol procedures
PPE — gloves	Single gloves	Double gloves mandatory	Double gloves mandatory
PPE — respiratory	Not routine	N95/FFP2 for aerosol-generating procedures	Respiratory protection per SOP
PPE — eye protection	When splash risk exists	Mandatory (goggles or face shield)	Mandatory
Room pressure	Not required	Recommended negative pressure	Negative pressure required
HEPA exhaust	Not required	Recommended for high-risk procedures	Required on exhaust air
Operator competency	General biosafety training	Documented procedure-specific training	Agent-specific and procedure-specific training
Supervision	Standard	Direct supervision for initial operations	Continuous oversight protocol
Institutional authorization	Standard risk assessment	Written risk assessment + IBC approval	Full IBC approval + regulatory notification
Access control	Limited access	Restricted access with documentation	Controlled access with an entry log

BSL-2+ is an operationally defined intermediate tier, not a formally codified biosafety level. Its application requires a documented risk assessment and authorization from the institutional biosafety committee. IBC, institutional biosafety committee; BSC, biosafety cabinet; PPE, personal protective equipment; SOP, Standard Operating Procedure. respiratory protection is given as the required protection level; the certified device follows the applicable regional standard (NIOSH N95/N99/N100 in the US/Canada; EN, 149 FFP2/FFP3 in the EU). N95 corresponds to FFP2; FFP3 (≥99% filtration) is recommended for the highest-risk aerosol-generating procedures.

Respiratory protection, regional standards. Respiratory-protection designations differ by jurisdiction, and assessors must apply the standard in force at their location. The N-series (N95/N99/N100; NIOSH 42 CFR 84) is used in the United States and Canada, whereas the European Union and most other jurisdictions follow EN 149 (FFP2/FFP3). For practical purposes an N95 respirator corresponds to an FFP2, while FFP3 (≥99% filtration efficiency) provides a higher level of protection, broadly comparable to N99/N100, and is recommended for the highest-risk aerosol-generating procedures, such as manipulation of high-titre RG3 agents transmissible by the airborne route. Throughout this framework, respiratory-protection requirements specify the level of protection needed; selection of the corresponding certified device is governed by the applicable regional standard.

Decontamination and waste inactivation. Control selection must additionally specify agent-appropriate decontamination and disinfection procedures for surfaces, equipment, and waste. Disinfectant choice must match the susceptibility of the agent: 70% ethanol and quaternary ammonium compounds are adequate for enveloped viruses and most vegetative bacteria, whereas non-enveloped viruses (e.g., adeno-associated virus) and bacterial spores require a validated virucidal/sporicidal agent (such as ≥0.5% sodium hypochlorite or an equivalent) applied at defined contact times (see Case Study 3, where standard ethanol is insufficient against non-enveloped AAV). Liquid and solid biological waste must be inactivated by autoclaving or validated chemical treatment before disposal, in accordance with institutional waste-management procedures.

#### 3.3.8 Step 8 — documentation and review (estimated time: 30–60 min)

Complete the full assessment record using the structured template (Supplementary Appendix S1). The written assessment must include: agent classification with source, procedure list with PSR assignments, modulating factors evaluation, likelihood–severity integration, BSL assignment with technical justification, and complete control specification. Submit to the IBC for review and approval prior to initiating laboratory work. Formally establish reassessment triggers: agent change, procedure modification, scale increase, incident or exposure event, new regulatory data, personnel change, or equipment failure. Set a next review date not exceeding 12 months from the approval date.

### 3.4 Validation approach

The initial validation of the PSR framework rests on three complementary forms of evidence that, taken together, constitute an appropriate foundation for a decision framework at this stage of development. First, content validity is established through the derivation of all framework components from structured synthesis of ten regulatory documents of recognized international authority (WHO, BMBL, CDC, INSST, EU Directives), ensuring that no classification criterion is introduced without regulatory grounding. Second, convergent validity is demonstrated by the consistent agreement between PSR-derived BSL assignments and established containment recommendations in BMBL (2020) and WHO (2020) across all six case studies. Third, coverage validity—the systematic representation of the framework’s decision space—is addressed through purposive case selection spanning all critical dimensions of the Agent × PSR matrix: RG1–3 classifications, Low–High PSR levels, procedure-driven escalation from a lower-RG agent, procedure-driven containment reduction from a higher-RG agent, identical-agent dual BSL assignment, and equivalent-outcome dual-technology comparison. This approach follows the validation trajectory established for analogous decision frameworks in occupational risk management, including Control Banding methodologies for chemical hazards, which achieved regulatory adoption on the basis of content and convergent validity prior to the availability of systematic empirical reliability data (Zalk and Nelson, 2008). Prospective inter-rater reliability assessment (target κ ≥ 0.60) across ≥10 qualified evaluators from ≥3 institutions represents the planned next validation step and is operationally supported by the digital implementation tool described in Supplementary Appendix S3.

The prospective validation protocol targets ≥10 qualified biosafety professionals from ≥3 institutions, assessing 10–15 representative scenarios—including at least two deliberately ambiguous cases—using the standardized template and the digital tool. Inter-rater agreement will be quantified using Cohen’s kappa (pairwise) and Fleiss’ kappa (multi-rater), with κ ≥ 0.60 (substantial agreement) as the operational threshold. Disagreement patterns will drive iterative refinement of classification criteria. The digital tool’s multi-user module provides the infrastructure for distributed, simultaneous assessment and automated kappa computation (Supplementary Appendix S3).

## 4 Results: framework development and validation approach

### 4.1 The PSR operational framework: development and design principles

Procedure classification matrices (Low/Moderate/High PSR) were developed by integrating: (a) aerosol generation potential, (b) volume thresholds (<10 mL, 10–100 mL, >100 mL), (c) concentration metrics (CFU/mL, PFU/mL, TU/mL, or MOI), and (d) equipment-specific factors (Pottage et al., 2022; Wurtz et al., 2016). These thresholds should be interpreted as operational heuristics rather than standardized regulatory cut-offs, as current international guidelines do not define universally accepted quantitative boundaries.

Implementation resources—assessment template, worked examples, digital tool, and cross-source alignment summary—are described in the Materials and Equipment section and provided in full in Supplementary Appendix S1–S4.

### 4.2 Procedure classification by PSR level

Procedures were classified into Low, Moderate, or High PSR categories based on aerosol generation potential, volume, and concentration thresholds (Table 6). This classification informs the estimated likelihood of exposure, independent of agent hazard (Pottage et al., 2022; Wurtz et al., 2016).

#### 4.2.1 Threshold derivation and epistemic status

Volume and concentration thresholds were derived from WHO (2020) guidance on small-versus large-scale operations, BMBL (2020) agent summary statements, and environmental monitoring studies reporting order-of-magnitude differences in bioaerosol generation between uncontained bench manipulations and Class II BSC operations (Pottage et al., 2022; Wurtz et al., 2016). These boundaries represent operational heuristics, not universally standardized regulatory cut-offs. Users should document professional judgment when classifying procedures at or near category boundaries, and treat boundary cases as candidates for the ≥2 modulating factors escalation rule (Step 4).

### 4.3 Modulating factors in PSR evaluation

Beyond procedure classification, operational context modulates actual risk through two conceptually distinct categories of factors: those that affect exposure probability (procedural modulators) and those that affect consequence severity (individual susceptibility modulators). Both categories inform the overall risk evaluation, but through different mechanisms and at different levels of analysis.

Procedural modulators (Table 7) act directly on the likelihood of exposure during a given manipulation, independently of agent hazard. The presence of two or more unfavorable procedural modulating factors triggers consideration for BSL elevation (e.g., BSL-2 to BSL-2+ or BSL-3) (Pottage et al., 2022; Wurtz et al., 2016; Pike, 1976).

#### 4.3.1 Escalation rule basis and weighting limitations

The ≥2 unfavorable modulating factors threshold is consistent with retrospective LAI analyses and with the categorical escalation logic of Control Banding frameworks for chemical hazards (Zalk and Nelson, 2008). The current rule applies equal weight to all nine procedural modulating factors. In practice, inadequate infrastructure or high aerosol generation may warrant greater escalation weight than procedural frequency alone; assessors should exercise and document professional judgment when the threshold is triggered by combinations of lower-impact factors. Weighted scoring derived from Delphi expert consensus or empirical exposure data is identified as a priority refinement.

Individual susceptibility modulators act on the severity of consequences should exposure occur, rather than on the probability of exposure. The primary factor is immune status: immunocompromised workers face substantially increased risk of severe outcomes following biological exposure. Because immune status does not alter the probability of exposure, it does not directly modify PSR-level assignment. Instead, it should be evaluated within the framework of individual worker risk assessment in accordance with occupational health surveillance programs (RD 664/1997, Directive 2000/54/EC) (España, 1997; European et al., 2000).

### 4.4 BSL assignment matrix: agent classification × PSR level

Throughout this framework, the notation RG3* denotes a biological agent derived from an RG3 parental organism that has been sufficiently attenuated—through documented genetic modification, replication incompetence, or validated inactivation—to warrant risk evaluation at a level below its parental classification. The designation BSL-2+ refers to an operationally defined containment tier: standard BSL-2 infrastructure augmented with systematically enhanced procedural controls—including mandatory use of Class II biosafety cabinets, double gloves, respiratory protection, eye protection, and documented operator competency—functionally equivalent to what BMBL describes as ‘BSL-2 with select BSL-3 practices’ and to the enhanced containment approach outlined in INSST guidance. Its application is always contingent on a written risk assessment and documented institutional authorization (International Organization for S, 2019).

The core operational output of PSR assessment is the Agent-PSR combination matrix (Table 8), which assigns biosafety levels based on integrated evaluation of agent hazard (Risk Group) and procedural likelihood (PSR). This matrix demonstrates that identical agents require different BSLs depending on procedure. BSL-2+ assignment for RG3 agents with attenuated transmissibility is permissible only under Low PSR conditions when all of the following are met: (a) documented risk assessment with explicit justification; (b) institutional biosafety approval; (c) implementation of enhanced containment controls (Class II BSC, double gloves, eye protection, and respiratory protection); and (d) documented operator competency. In all other scenarios, BSL-3 remains the default containment level.

Table 9 provides a detailed breakdown of the minimum containment requirements distinguishing BSL-2, BSL-2+, and BSL-3 tiers across ten key operational dimensions.

## 5 Illustrative applications

Application of the 8-step PSR protocol to six representative laboratory scenarios demonstrates the framework’s capacity for systematic decoupling of BSL assignment from automatic RG classification: identical agents assessed under different procedural conditions yield different, independently justified containment levels. Table 10 consolidates all six BSL assignments. Cases 1 and 2 are presented in full narrative detail below; Cases 3–6 follow in structured abbreviated form. Complete assessments for all cases are provided in Supplementary Appendix S2.

**TABLE 10:** Procedure-Specific Risk (PSR) Decision Matrix across representative laboratory scenarios.

Case	Agent (classification)	Key procedures	Max PSR	Critical modulating factors	Likelihood	Severity	Risk level	BSL assigned	PSR decision logic
1	Lentiviral vector (HIV-1 derived, RG2)	Transfection, ultracentrifugation, high-titer concentration	High	High titer, aerosol, non-sealed centrifugation	Very Likely	Moderate	High	BSL-2+	Aerosol + concentration override
2	M. tuberculosis (RG3)	Plate culture, subculture in BSC-II	Low	Low volume (<1 mL), BSC-II containment, high environmental stability, daily frequency	Rare	High	Moderate	BSL-2+ (BSL-3 preferred for aerosol-generating procedures)	Low PSR enables proportionate reduction from default BSL-3 under strict conditions
3	Recombinant AAV2 (RG2, precautionary)	Large-scale production, ultracentrifugation	High	Large volume, high stability	Very Likely	Moderate	High	BSL-2+	Scale + persistence
4	E. coli BL21 (RG1)	High-density sonication	High	Extreme concentration, aerosol generation	Likely	Low	Moderate	BSL-1 (standard); BSL-2 practices (sonication step only)	Procedure-driven
5	HIV-1 (wild-type, RG3)	Routine cell infection/Virus concentration	Moderate/High	No airborne route; low vol (A)/high titer + aerosol risk (B)	Possible/Very Likely	Moderate	Moderate (A)/High (B)	BSL-2+/BSL-3	Same RG3 agent, two BSL outcomes driven by procedure
6A	CRISPR lentiviral delivery (RG2)	Viral production and transduction	High	Viral titers, aerosol risk	Very Likely	Moderate	High	BSL-2+	Vector risk
6B	CRISPR RNP delivery (RG1)	Electroporation (closed system)	Low	Small volume, non-replicative	Rare	Low	Low	BSL-1	Minimal exposure

PSR classification and likelihood categories are semi-quantitative and intended to support structured decision-making. Final BSL assignment requires institutional validation and alignment with applicable regulatory frameworks.

### 5.1 Expected outputs by scenario type

For RG1 agents under standard conditions (Low–Moderate PSR), the framework confirms BSL-1; high-aerosol-generating steps (e.g., sonication ≥10^9^ CFU/mL) yield BSL-2 practices restricted to that step only (Case Study 4). For RG2 agents, Moderate or High PSR produces BSL-2 or BSL-2+ depending on procedural parameters and modulating factors; high-titer viral vector production with ultracentrifugation and ≥2 unfavorable modulating factors consistently triggers BSL-2+ (Case Studies 1, 3, 6A). For RG3 agents, the framework produces the most operationally significant differentiation: routine low-volume BSC-contained manipulations may yield BSL-2+ under strict conditions (Case Studies 2 and 5A), while high-PSR procedures with the same agent mandate BSL-3 (Case Study 5B) — a divergence that static RG→BSL correlation cannot produce. The full decision logic for each case is presented in Table 10 and in the case studies below.

### 5.2 Representative case studies

Six case studies were purposively selected to demonstrate maximum procedural variation across diverse biological agents and procedures, representing RG1–3 classifications, low-to-high PSR procedures, and viral/bacterial/GMO systems. BSL-4 scenarios were not included as illustrative cases given the extremely limited number of institutions operating at this containment level and the highly agent-specific nature of BSL-4 assignments, which offer limited generalizable procedural variation. BSL-3 is represented indirectly through Case Study 5B, where high-titer ultracentrifugation of wild-type HIV-1 yields a BSL-3 assignment, and through Case Study 2, where the framework explicitly identifies BSL-3 as the default for RG3 agents outside the narrow conditions permitting BSL-2+. Cases 1 and 2 are presented in extended narrative form with full step-by-step detail; Cases 3–6 follow the same structured format in a parallel presentation. All six complete structured assessments are provided in Supplementary Appendix S2.

#### 5.2.1 Case study 1: third-generation lentiviral vector production (HIV-1-based)

Agent: Third-generation lentiviral vector (HIV-1 backbone, replication-defective). Classification: RG2 (documented attenuation).

Procedures: HEK293T transfection → Moderate PSR; supernatant collection → Moderate PSR; filtration → Moderate PSR; ultracentrifugation (25,000 rpm) → High PSR; viral pellet resuspension → High PSR.

Modulating factors: Volume 50–100 mL (moderate), final concentration 10^8^–10^9^ TU/mL (high), weekly frequency (high), low environmental stability (favorable), operator >2 years experience (favorable). Four unfavorable procedural modulating factors (volume, concentration, frequency, non-sealed centrifugation) — escalation rule triggered.

BSL Assignment: RG2 + High PSR → BSL-2+. Despite RG2 classification, high-titer production and ultracentrifugation steps generate significant aerosol risk compounded by four unfavorable modulating factors. Required key controls include: mandatory Class II BSC for all open manipulations, sealed centrifuge rotors or safety cups, N95/FFP2 respiratory protection during centrifugation and pellet resuspension, double gloves, negative pressure (recommended), and documented operator competency assessment prior to initiating production.

#### 5.2.2 Case study 2: mycobacterium tuberculosis diagnostic culture

Agent: M. tuberculosis (RG3). Classification maintained due to high pathogenicity and potential for airborne transmission.

Procedures: Plate seeding on solid medium in BSC-II → Low PSR; incubation 3–6 weeks (sealed); colony observation (sealed plate) → Low PSR; subculture in BSC-II → Low PSR.

Modulating factors: Volume <1 mL (favorable), daily frequency (unfavorable), high environmental stability (unfavorable). Only 1 unfavorable procedural modulating factor—escalation rule not triggered.

BSL Assignment: RG3 + Low PSR → BSL-2+ (permissible when all work performed in BSC-II, N95 respirators used, institutional TB control program exists, and no high-PSR procedures are performed). BSL-3 preferred for research involving large volumes or aerosol-generating procedures.

#### 5.2.3 Case study 3: large-scale recombinant AAV2 production for gene therapy

Triple transfection and iodixanol gradient ultracentrifugation at 500 mL per run, final titer 10^12^ particles/mL. Four unfavorable modulating factors, with extreme environmental stability (resistant to heat, detergents, and pH extremes) as the critical driver. Although nominally RG2, AAV’s non-enveloped structure and environmental persistence create contamination risk disproportionate to its classification, mandating BSL-2+ with dedicated negative-pressure room, sealed ultracentrifuge rotors, and 0.5% bleach decontamination—standard ethanol being insufficient against non-enveloped AAV. Some institutional protocols recommend BSL-3 for this scale; institutional review is advised. Full assessment: Supplementary Appendix S2, Example C.

#### 5.2.4 Case study 4: E. coli BL21 bacterial pellet sonication for recombinant protein

Standard culture and purification steps remain BSL-1 throughout. The sonication step (6 cycles × 30 s, open vessel, 10^9^–10^10^ CFU/mL) generates three unfavorable modulating factors—extreme concentration, open configuration, weekly frequency—and triggers the escalation rule for that step alone, requiring BSL-2 practices. This case illustrates that the same laboratory, same organism, and same experiment may require two containment levels for different procedural steps. Primary risk-elimination measure: substitution with a cup-horn sonicator (Pottage et al., 2022; Ravnholdt et al., 2025; Wurtz et al., 2016). Full assessment: Supplementary Appendix S2, Example D.

#### 5.2.5 Case study 5: HIV-1 procedural risk stratification—Same agent, two BSL outcomes

Agent: HIV-1 (wild-type, replication-competent, RG3). Critical favorable trait: strictly parenteral and mucosal transmission—no airborne route—with effective PEP and antiretroviral therapy available.

Sub-protocol A—Routine cell infection (5–10 mL, Moderate PSR, 1 unfavorable factor): absence of airborne transmission, low working volume, and complete BSC containment reduce procedural exposure probability below the BSL-3 threshold → BSL-2+, consistent with BMBL (2020) and WHO (2020) guidance for routine HIV research in non-clinical settings (Centers for Disease C et al., 2020; World Health, 2020).

Sub-protocol B—High-titer ultracentrifugation (50–200 mL at 10^7^–10^8^ TCID_50_/mL, High PSR, 3 unfavorable factors): aerosol-generating centrifugation at very high viral titers cannot be safely managed under BSL-2 infrastructure regardless of procedural controls → BSL-3, requiring directional airflow, HEPA-filtered exhaust, sealed centrifuge rotors, and negative pressure (Centers for Disease C et al., 2020; World Health, 2020; Pottage et al., 2022; Ravnholdt et al., 2025; Wurtz et al., 2016).

This divergence—two distinct BSL assignments for the same RG3 agent—is the most operationally significant output of the PSR framework and cannot be produced by static RG→BSL correlation. Full assessment: Supplementary Appendix S2, Example E.

#### 5.2.6 Case study 6: CRISPR-Cas9 delivery—lentiviral vector vs. RNP electroporation

Both sub-protocols achieve equivalent CRISPR-Cas9 genome-editing outcomes in human cell lines; biosafety requirements diverge exclusively due to procedural risk profile.

Sub-protocol A—Lentiviral delivery (third-generation, replication-defective, RG2): ultracentrifugation-based concentration at 10^7^–10^9^ TU/mL, 2–3 unfavorable modulating factors, escalation rule triggered → BSL-2+. Replication-defective status prevents escalation to BSL-3, but aerosol-generating concentration steps mandate enhanced containment beyond standard BSL-2 (Bouard et al., 2009; Kotterman et al., 2015; Naldini, 1998; Sinn et al., 2005; Wang et al., 2019; Adli, 2018; Doudna and Charpentier, 2014).

Sub-protocol B—RNP electroporation (pre-assembled Cas9–gRNA complexes, RG1): electroporation in closed cuvette, volumes <5 mL, no unfavorable modulating factors → BSL-1 (enhanced electrical safety practices recommended). Elimination of replication-capable intermediates and absent environmental persistence reduce exposure probability to negligible (Adli, 2018; Doudna and Charpentier, 2014; Ran et al., 2013; Wang et al., 2016). Full assessment: Supplementary Appendix S2, Example F.

### 5.3 Advantages of the protocol

The PSR framework offers three principal operational advantages over agent-centric classification. First, it enables proportionate containment: procedures that generate low exposure likelihood, even with higher-RG agents, are not systematically over-contained, reducing resource expenditure without compromising personnel protection. Second, it produces documented, auditable decisions: the written assessment record and IBC approval trail satisfy regulatory documentation requirements under RD 664/1997, Directive 2000/54/EC, and equivalent national legislation. Third, the framework’s modular architecture accommodates emerging laboratory technologies—including CRISPR-based platforms, synthetic biology systems, and novel viral vectors—whose risk profiles are not fully captured by existing agent classification schemes, as demonstrated by the divergent BSL assignments for lentiviral versus RNP delivery of CRISPR-Cas9 (Case Studies 6A and 6B).

### 5.4 Limitations and possible pitfalls

Several implementation challenges are anticipated. The framework requires procedure-specific biosafety expertise to complete Steps 2–4 accurately; assessors without training in aerosol generation characterization or pathogen concentration estimation may misclassify PSR levels, particularly at the Low/Moderate boundary.

Crucially, successful implementation of the PSR framework relies on the active involvement of the institutional biosafety office. The structured template and digital tool standardize the evaluation logic, but they do not replace the specialized expertise the assessment requires: accurately characterizing aerosol-generation potential, volume thresholds, and the impact of modulating factors (Steps 2–4) depends on training that is not readily acquired by junior researchers or general laboratory personnel. The framework should therefore not be construed as a standalone self-assessment instrument for independent use by junior staff, reinforcing that it is not a lay self-assessment application. It is better understood as a structured communication interface operating within the laboratory’s existing oversight chain. The bench researcher, most familiar with the manipulations, maps the technical workflow and operational parameters; the Principal Investigator supervises this characterization within its scientific and experimental context, ensuring it reflects the work as actually planned; and the designated Biosafety Officer (BSO) supplies the expertise needed to validate the exposure-likelihood estimates, arbitrate borderline classifications (particularly at the Low/Moderate PSR boundary), and consolidate the proposed BSL assignment. The biosafety office thus performs an essential mediating role, reviewing and validating every PSR assessment before submission to the IBC, which retains final authorization. Under this model, standardized decision-making remains grounded in expert oversight, and the expertise requirement is met by making specialist review a defined step in the workflow rather than an optional one.

The ≥2 unfavorable modulating factors escalation rule applies equal weight to all nine procedural modulators. In practice, certain factors—particularly inadequate infrastructure and high aerosol generation—may carry greater escalation weight than procedural frequency alone. Assessors should exercise professional judgment when the escalation rule is triggered by combinations of lower-impact factors, and document their reasoning explicitly in the written assessment.

The quantitative thresholds in Tables 6, 7 represent operational heuristics, not universally standardized regulatory cut-offs. Their applicability across diverse biological systems requires empirical evaluation through the prospective validation study described in the Methods section.

The BSL-2+ designation is an operationally defined intermediate tier, not a formally codified biosafety level. Its assignment is always contingent on written IBC authorization. Institutions operating under regulatory frameworks that do not recognize intermediate containment tiers should default to BSL-3 when the framework produces a BSL-2+ output.

Finally, the framework’s scope is limited to contained laboratory use in diagnostic and research settings. Industrial-scale production, ABSL, fieldwork, and biosecurity threat assessment require additional evaluation frameworks not addressed by this protocol.

## 6 Discussion

The PSR framework presented here operationalizes a principle that international biosafety guidance has endorsed but not yet standardized: that containment decisions should be driven by the exposure a given procedure generates, not by agent taxonomy alone. By showing convergence across ten regulatory sources, despite their terminological heterogeneity, and translating it into a stepwise decision model, this work moves procedure-specific risk from an implicit regulatory expectation to an explicit, reproducible protocol.

The jurisdictional scope is deliberately European and North American, but the five convergence principles identified (multifactorial integration, rejection of automatic RG–BSL correlation, dynamic revisability, mandatory documentation, and proportionate containment) are consistent with globally recognized risk-based biosafety paradigms (Kimman et al., 2008; Centers for Disease C et al., 2020; World Health, 2020). In practice, this shifts containment decisions from categorical agent classification toward an exposure-informed model in which the same organism may require different containment depending on how it is handled.

### 6.1 Conceptual shift from static to dynamic risk assessment

The PSR framework reflects a broader shift in how biological risk is understood in laboratory settings: risk is treated not as a fixed property of the agent but as something that varies with how the agent is handled. The fundamental equation Risk = Hazard × Likelihood, explicitly formalized in CDC (2024) and implicit in WHO/BMBL frameworks, separates agent-dependent hazard (pathogenicity, virulence, treatment availability) from procedure-dependent likelihood (aerosol generation, volume, operator competency). This separation enables more proportionate and evidence-informed control selection, which is difficult to achieve within rigid RG–BSL schemes ((Kimman et al., 2008), (National Research, 1989), (Pottage et al., 2022; Ravnholdt et al., 2025; Wurtz et al., 2016), (Pike, 1976)).

The six case studies quantify this shift in operational terms. The most illustrative instance is Case Study 5: wild-type HIV-1 (RG3) warrants BSL-2+ for routine low-volume cell infection in a Class II BSC—where the absence of airborne transmission and complete containment dominate the exposure equation—but BSL-3 for high-titer ultracentrifugation of the same strain. No static RG→BSL rule can produce this discriminative output. HIV-1 is exactly the kind of non-airborne Group 3 agent that European Directive 2000/54/EC recognizes as posing a limited infection risk (Section 1.1), giving this BSL-2+ assignment a direct legislative basis. The epidemiological basis for this approach is well-established: uncontained bench manipulations generate substantially higher bioaerosol concentrations than equivalent BSC-contained procedures (Pottage et al., 2022; Ravnholdt et al., 2025), and retrospective laboratory-acquired infection surveys indicate that aerosol-generating steps are disproportionately represented in exposure events (Wurtz et al., 2016; Pike, 1976).

### 6.2 Operational advantages and implementation challenges

PSR assessment delivers three operational advantages over agent-centric classification. First, proportionate containment: procedures with low exposure likelihood are not systematically over-contained, reducing resource expenditure without compromising protection. Second, documented, auditable decisions: the written assessment record and IBC approval trail satisfy regulatory documentation requirements under RD 664/1997, Directive 2000/54/EC, and equivalent national legislation (España, 1997; European et al., 2000; Centers for Disease C et al., 2020; World Health, 2020; International Organization for S, 2019). Third, technology-neutral adaptability: the framework’s modular design accommodates emerging platforms—viral vectors, CRISPR-Cas9 systems (Adli, 2018; Doudna and Charpentier, 2014; Ran et al., 2013; Wang et al., 2016; Khalil and Collins, 2010; Pickar-Oliver and Gersbach, 2019), synthetic biology (Khalil and Collins, 2010; Evans and Selgelid, 2015; Gómez-Tatay and Hernández-Andreu, 2019) — whose risk profiles evolve faster than agent classification schemes can. Case Study 6 illustrates this directly: two CRISPR delivery modalities achieving identical editing outcomes receive BSL-2+ and BSL-1 respectively, because biosafety follows procedural risk, not biotechnology category. The digital tool supports institutional analytics and inter-rater reliability computation (Supplementary Appendix S3).

Implementation challenges include: (a) requirement for specialized biosafety expertise, (b) initial resource investment in SOP development and personnel training, (c) institutional culture change from prescriptive to performance-based regulation, and (d) absence of standardized international terminology impeding cross-jurisdictional harmonization. The terminological heterogeneity documented in Table 4 suggests an opportunity to establish international consensus on “Procedure-Specific Risk (PSR)” as a unified nomenclature, potentially through WHO or ISO standardization processes.

### 6.3 Limitations, scope, and validation roadmap

First, this work represents a narrative synthesis and the development of a conceptual framework, rather than a systematic review complying with PRISMA or an empirical study with primary data collection. The framework should be interpreted as a structured decision-support framework rather than a predictive risk model. These constraints are shared by integrative methodological frameworks across risk disciplines, including the Control Banding approach for chemical hazards, which followed an analogous trajectory of expert-derived deployment followed by progressive empirical validation (Greenhalgh et al., 2018). Notably, all six case study BSL assignments are consistent with established containment recommendations in BMBL (2020) and WHO (2020), supporting the convergent validity of the framework’s decision logic.

Second, the case studies prioritize well-characterized, relatively clear-cut cases. Deliberately ambiguous situations—such as novel engineered organisms without established RG classifications, procedures with uncertain aerosol-generating potential, or emerging biotechnologies at the intersection of multiple regulatory frameworks—pose important validation challenges not fully addressed here.

Third, the jurisdictional scope is deliberately delimited to European and North American regulatory frameworks. Key Asia-Pacific frameworks—including China’s Biosafety Law (2020), Japan’s Cartagena Act (2003, revised 2019), and Australia’s Gene Technology Act (2000)—incorporate risk-based principles broadly compatible with the five PSR convergence principles identified here and are identified as the primary jurisdictional expansion priority for subsequent work.

Fourth, the scope is limited to laboratory biosafety in diagnostic and research settings. Industrial-scale production, ABSL, fieldwork, mobile laboratory operations, and biosecurity considerations lie beyond the scope of this article. Environmental risk assessment, including potential ecological impacts associated with accidental release of genetically modified organisms, is likewise outside the present scope: it is governed by distinct instruments (the Cartagena Protocol and the contained-use provisions of Directive 2009/41/EC). Adapting PSR principles to environmental biosafety represents an important future extension.

Fifth, comprehensive biosafety programs require integration of PSR with complementary risk management components—medical surveillance, waste management, emergency response planning, and incident investigation—which lie outside the scope of this methodological synthesis. The prospective multi-institutional validation protocol described in Section 3.3 provides the infrastructure for the next step: quantifying inter-rater agreement across diverse institutional contexts. Successful completion would position the PSR framework as an evidence-based standard for procedure-specific BSL assignment. Priority areas for subsequent development include weighted scoring for modulating factors informed by Delphi expert consensus, empirical validation of volume and concentration thresholds through environmental monitoring studies, and jurisdictional expansion to Asia-Pacific regulatory frameworks—identified as a priority in the document selection phase but outside the scope of this initial synthesis.

### 6.4 Conclusions

The PSR framework operationalizes a reframing of laboratory biological risk—from a static agent property to a dynamic agent-procedure parameter—and translates the convergence of ten regulatory frameworks on five core principles into a structured, reproducible 8-step method for BSL assignment. The six case studies show how identical agents warrant different containment levels depending on procedural exposure probability, illustrating the practical limitations of purely agent-based classification and the operational value of decoupling BSL from RG. Practical adoption requires no structural regulatory modification: the framework is compatible with existing institutional biosafety programs and supported by the assessment template, worked examples, and bilingual digital tool in Supplementary Appendix S1–S3.

As bioengineering capabilities expand into domains such as hierarchical metabolic engineering, open-source synthetic biology, and advanced gene-editing platforms, the risk profiles of laboratory procedures will increasingly diverge from those predicted by parental-organism classification alone. Frameworks such as PSR, grounded in dynamic exposure-informed evaluation, provide a structured basis for adapting biosafety governance to these evolving operational realities (Evans and Selgelid, 2015; Gómez-Tatay and Hernández-Andreu, 2019; Chai et al., 2025; Millett et al., 2019).

## Data Availability

The original contributions presented in the study are included in the article/Supplementary Material, further inquiries can be directed to the corresponding author.
