# Supplementary material for: An 8-step procedure-specific risk framework enables reproducible biosafety level assignment beyond agent-based classification
Source: Front Bioeng Biotechnol. 2026 Jul 17;14:1879247. doi: 10.3389/fbioe.2026.1879247 (PMC13423866; doi:10.3389/fbioe.2026.1879247)
Supplement: Supplementary file 1 [file Table1.docx]

**Supplementary Appendix S1**

**PSR Assessment Template with Integrated Methodology Checklist**

This appendix provides a structured, editable template for conducting procedure-specific biological risk assessments using the 8-step PSR framework. Each section corresponds to one framework step. A methodology verification checklist is included at the end (Section 10) to ensure completeness before institutional review.

## Section 1 — Project Information

| **Project Title:** |  |
| --- | --- |
| **Principal Investigator:** |  |
| **Institution / Department:** |  |
| **Assessment Date:** |  |
| **Assessor Name:** |  |
| **Next Review Date:** |  |
| **Protocol Number:** |  |

## Section 2 — Agent Characterization (Step 1)

**Biological Agent Name:** ___________________________

**Taxonomic Classification:** ___________________________

**Risk Group Classification:**

☐ RG1 — Low individual and community risk

☐ RG2 — Moderate individual risk, low community risk

☐ RG3 — High individual risk, low community risk

☐ RG4 — High individual and community risk

**Reference Source:**

☐ WHO ☐ BMBL ☐ EU Directive 2000/54/EC ☐ INSST ☐ Other: ________

GMO Status: ☐ Yes ☐ No If GMO → describe modification, parental organism, replication competence:

_________________________________________________________________________

**Key Biological Traits:**

| **Route of Transmission:** |  |
| --- | --- |
| **Environmental Stability:** |  |
| **Infectious Dose:** |  |
| **Treatment Available:** |  |
| **Additional Hazards:** |  |

## Section 3 — Procedure Characterization (Step 2)

List all laboratory manipulations with volumes, concentrations, and aerosol-generation potential:

| **Procedure Step** | **Volume** | **Conc.** | **Aerosol** | **Equipment** | **Freq.** |
| --- | --- | --- | --- | --- | --- |
|  |  |  |  |  |  |
|  |  |  |  |  |  |
|  |  |  |  |  |  |
|  |  |  |  |  |  |
|  |  |  |  |  |  |
|  |  |  |  |  |  |

**Highest-Risk Procedural Step:** ___________________________

## Section 4 — PSR Classification (Step 3)

| **Criterion** | **Low PSR** | **Moderate PSR** | **High PSR** |
| --- | --- | --- | --- |
| **Aerosol** | Minimal / none | Moderate potential | High (centrifugation, sonication) |
| **Volume & Conc.** | <10 mL; <10⁶ CFU/PFU/mL | 10–100 mL; 10⁶–10⁸ | >100 mL; >10⁸ CFU/PFU/mL |

*For representative procedures for each PSR level, refer to Table 6 in the main article.*

Assigned PSR Level: ☐ Low ☐ Moderate ☐ High

**Rationale:** ___________________________

## Section 5 — Modulating Factors Evaluation (Step 4)

Evaluate the nine procedural modulating factors below (Table 7 of the main article). An additional row for individual susceptibility is included for documentation purposes; this factor affects the severity of consequences and is not counted toward the ≥2 escalation rule (see Section 3.3 of the main article).

| **Factor** | **Risk-Increasing Condition** | **Present?** | **Mitigation** |
| --- | --- | --- | --- |
| **Volume** | >100 mL |  |  |
| **Concentration** | >10⁸ CFU/PFU/mL |  |  |
| **Frequency** | Daily/weekly |  |  |
| **Aerosol Generation** | High (sonication, vortex) |  |  |
| **Environmental Stability** | High (AAV, spores) |  |  |
| **Operator Experience** | <6 months |  |  |
| **Supervision** | None / insufficient |  |  |
| **Infrastructure** | Inadequate BSL |  |  |
| **Incident History** | Previous exposures |  |  |
| **Individual Susceptibility** | Immunocompromised personnel or the absence of available vaccination |  |  |

Note: Individual susceptibility affects the severity of consequences rather than the likelihood of exposure and should not be counted toward the ≥2 unfavorable factors escalation rule. It must be addressed through occupational health measures (Step 8).

**Decision Rule: Presence of ≥2 unfavorable factors triggers containment escalation.**

Number of Unfavorable Factors: _____ Escalation Triggered? ☐ Yes ☐ No

## Section 6 — Likelihood × Severity Integration (Step 5)

Severity: ☐ Low (RG1) ☐ Moderate (RG2) ☐ High (RG3) ☐ Extreme (RG4)

Likelihood: ☐ Rare ☐ Possible ☐ Likely ☐ Very Likely

*The following guidance relates PSR classification to the likelihood categories used in the risk matrix. Modulating factors (Section 5) may shift the likelihood within or beyond these ranges:*

| **PSR Level** | **Default Likelihood** | **With ≥2 unfavorable factors** |
| --- | --- | --- |
| **Low** | Rare – Possible | Possible – Likely |
| **Moderate** | Possible – Likely | Likely – Very Likely |
| **High** | Likely – Very Likely | Very Likely |

These correspondences are indicative. The assessor should exercise professional judgment, considering the specific modulating factors profile documented in Section 5.

| **Severity ↓ / Likelihood →** | **Rare** | **Possible** | **Likely** | **Very Likely** |
| --- | --- | --- | --- | --- |
| **Low** | Negligible | Low | Low | Moderate |
| **Moderate** | Low | Moderate | Moderate | High |
| **High** | Moderate | High | High | Very High |
| **Extreme** | High | Very High | Very High | Critical |

**Overall Risk Determination:** ___________________________

## Section 7 — Biosafety Level Assignment (Step 6)

Assigned BSL: ☐ BSL-1 ☐ BSL-2 ☐ BSL-2+ ☐ BSL-3 ☐ BSL-4

**Technical Justification (required): Reference RG, PSR level, modulating factors, and risk matrix result.**

_________________________________________________________________________

_________________________________________________________________________

## Section 8 — Control Measures (Step 7)

**Engineering Controls:**

☐ Class II BSC (A2/B2) ☐ Negative pressure ☐ Sealed centrifuge rotors ☐ HEPA filtration ☐ Autoclave ☐ Self-closing doors ☐ Other: ______

**PPE:**

☐ Single gloves ☐ Double gloves ☐ Eye protection ☐ Respiratory (N95/FFP2) ☐ Disposable gown ☐ Lab coat ☐ Other: ______

**Administrative Controls:**

☐ SOPs approved ☐ Training documented ☐ Access restricted ☐ Medical surveillance ☐ Incident reporting ☐ Waste plan ☐ Emergency plan ☐ Other: ______

☐ Occupational health evaluation/task reassignment (if applicable)

☐ Vaccination review (if available)

## Section 9 — Documentation and Review (Step 8)

**Reassessment Triggers:**

☐ New agent ☐ Procedure modified ☐ Incident/exposure ☐ Scale increase ☐ New regulatory data ☐ Personnel change ☐ Equipment failure

**Next Review Date:** ___________________________

| **Role** | **Name / Signature / Date** |
| --- | --- |
| Principal Investigator / Assessor |  |
| Biosafety Officer |  |
| IBC Chair |  |

## Section 10 — Methodology Verification Checklist

Verify all elements before submission to the Institutional Biosafety Committee:

☐ RG assignment cites authoritative source (WHO, BMBL, national legislation)

☐ GMO/synthetic evaluation: modifications, parental organism, replication competence specified

☐ Biological traits (pathogenicity, transmission, stability) characterized

☐ Highest-risk procedural step identified

☐ Working volumes and pathogen titers estimated with quantitative metrics

☐ Procedures classified by PSR level (Low/Moderate/High) using a standardized matrix

☐ All procedural modulating factors evaluated; ≥2 unfavorable factors escalation rule applied

☐ Individual susceptibility assessment documented (if immunocompromised personnel involved)

☐ Likelihood × Severity matrix completed; overall risk level determined

☐ BSL assigned with proportionate containment rationale

☐ Written technical justification provided

☐ Assessment reviewed/approved by Institutional Biosafety Committee

☐ Reassessment triggers formally established

Checklist completed by: ____________________ Date: ____________

*Supplementary Appendix S1. Operational template with integrated methodology verification checklist for conducting procedure-specific biological risk assessments. Implements the complete 8-step PSR framework in a structured, editable format for direct institutional use.*

**Supplementary Appendix S2**

**Worked Examples: PSR Framework Applied to Representative Laboratory Scenarios**

Six worked examples demonstrate the PSR framework across a range of biological agents (RG1–3), procedural risk levels (Low–High), and biological system types (viral vectors, bacteria, GMOs, gene-editing platforms). Examples A–F correspond to Case Studies 1–6 in Section 5.2 and Table 10 of the main article (Example E-A/E-B = Case Study 5A/5B; Example F-A/F-B = Case Study 6A/6B). Examples A and B are presented in full step-by-step detail. Examples C–F are presented in standardized abbreviated form covering all assessment-critical sections. All six follow the template structure of Supplementary Appendix S1 and are provided here to enable full reproducibility evaluation of the framework’s decision logic. Generic control checklists are summarized for brevity.

# Example A — High-Titer Lentiviral Vector Production (BSL Escalation)

Scenario: A research laboratory produces third-generation self-inactivating lentiviral vectors (HIV-1 backbone) for CRISPR-Cas9 delivery. The protocol involves large-scale transfection, viral supernatant collection, ultracentrifugation-based concentration, and aliquoting of high-titer stocks.

| **PI:** | Dr. Jane Smith | **Date:** | January 15, 2025 |
| --- | --- | --- | --- |
| **Institution:** | University Research Institute | **Protocol:** | IBC-2025-001 |

### Agent (Step 1)

Third-generation SIN lentiviral vector (pLenti-CRISPR). Parental: HIV-1 (RG3). Genetic modification: split-genome packaging, deletions of gag/pol/env/tat/rev/vif/vpr/vpu/nef, replication-incompetent (recombination risk <10⁻⁸). Replication incompetence experimentally validated through the absence of detectable viral propagation in permissive cell lines. Classification: RG2 (documented attenuation per BMBL Appendix B). Note: classification as RG3* (replication-defective GMO derived from RG3) would yield the same BSL-2+ assignment via Table 8 of the main article. Environmental stability: low (enveloped, sensitive to detergents).

### Procedures (Step 2)

| **Procedure** | **Volume** | **Concentration** | **Aerosol** | **PSR** | **Freq.** |
| --- | --- | --- | --- | --- | --- |
| HEK293T transfection (BSC) | 95 mL | Plasmid DNA | Minimal | Moderate | 2×/wk |
| Supernatant harvest (BSC) | 95 mL | 10⁵ TU/mL | Minimal | Moderate | 2×/wk |
| Filtration 0.45 µm (BSC) | 95 mL | Low titer | Low | Moderate | 2×/wk |
| Ultracentrifugation (SW32Ti, 25K rpm, non-sealed) | 4×30 mL | Pre-conc. | HIGH | HIGH | Weekly |
| Pellet resuspension (BSC) | 500 µL | 10⁸ TU/mL | Moderate | HIGH | Weekly |
| Aliquoting (multichannel) | 50 µL | 10⁸ TU/mL | Low-Mod | Moderate | Weekly |

**Highest-risk step: Ultracentrifugation at 25,000 rpm for 90 min without sealed safety cups.**

### PSR Classification & Modulating Factors (Steps 3–4)

Maximum PSR: HIGH (ultracentrifugation + resuspension steps).

**Unfavorable modulating factors (4 unfavorable procedural factors):**

• Procedural frequency (2×/week production)

• Scale of operation (120–150 mL per run)

• Infrastructure limitation (ultracentrifuge lacks sealed rotor — budget constraint)

• High-titer product (10⁸ TU/mL = 1000× concentration)

Favorable factors (3): low environmental stability, >2 years of operator experience, and direct supervision.

**≥2 unfavorable factors → escalation rule triggered.**

### Risk Integration & BSL Assignment (Steps 5–6)

Severity: Moderate (RG2, replication-defective). Likelihood: Very Likely (High PSR + 4 unfavorable factors). Risk matrix: Moderate × Very Likely = HIGH.

**BSL Assignment: BSL-2+.**

### Justification

Although classified as RG2 and replication-defective, the ultracentrifugation without sealed cups generates a significant aerosol risk compounded by a high titer (10⁸ TU/mL). Four unfavorable modulating factors warrant escalation. Standard BSL-2 is insufficient; BSL-3 is disproportionate given replication-defective status and low environmental persistence. BSL-2+ with respiratory protection during centrifugation, mandatory BSC-II for all steps, sealed rotors when available, and negative pressure provides proportionate containment, consistent with BMBL guidance for aerosol-generating procedures.

### Key Controls (Step 7, summary)

Engineering: mandatory BSC-II all open manipulations; negative pressure (−0.03" H₂O); sealed centrifuge buckets (to acquire); HEPA exhaust; autoclave within barrier. PPE: double gloves during centrifugation/resuspension; goggles mandatory; N95/FFP2 during aerosol steps; disposable gown. Administrative: SOP-LV-2025-001 approved; competency assessment completed; keycard access; medical surveillance; chemical inactivation before autoclaving; spill kit posted.

### Review Triggers (Step 8)

Sealed rotor acquisition (reassess BSL); new personnel; scale >200 mL/run; incident; annual review (Jan 2026). Signatures: PI (Dr. J. Smith), BSO (Dr. J. Doe), IBC (Dr. S. Johnson) — all Jan 2025.

# Example B — Proportionate Containment Reduction for RG3 Agent (Inactivated M. tuberculosis)

Scenario: A molecular diagnostics laboratory develops a real-time PCR assay for M. tuberculosis using exclusively heat-inactivated clinical specimens and purified nucleic acids. No viable M. tuberculosis is cultured in this facility.

| **PI:** | Dr. Maria Garcia | **Date:** | February 1, 2025 |
| --- | --- | --- | --- |
| **Institution:** | Clinical Diagnostics Lab, Infectious Disease Center | **Protocol:** | BSC-2025-008 |

### Agent (Step 1)

Mycobacterium tuberculosis complex — handled exclusively as heat-inactivated specimens (56°C, 30 min) and purified genomic DNA. Parental organism: RG3 (WHO, BMBL). Not a GMO. Key trait: airborne transmission applies only to viable organisms — NOT applicable to inactivated material. Environmental stability: high (viable) but low (inactivated, no viable organisms). Infectious dose: 1–10 bacilli by inhalation — NOT applicable to inactivated material. The specific inactivation protocol (e.g., 56°C for 30 min) must be **internally validated** using culture-negative confirmation for the specific equipment and sample matrix before authorizing containment reduction.

### Procedures (Step 2)

| **Procedure** | **Volume** | **Conc.** | **Aerosol** | **PSR** | **Freq.** |
| --- | --- | --- | --- | --- | --- |
| Receipt of inactivated specimens (sealed tubes) | 2–5 mL | N/A (inact.) | None | LOW | Daily |
| DNA extraction (QIAcube, closed system, BSC) | 200 µL | N/A | Minimal | LOW | Daily |
| PCR setup (pipetting in BSC) | 25 µL | N/A | Minimal | LOW | Daily |
| Thermal cycling (sealed PCR tubes) | Sealed | N/A | None | LOW | Daily |
| Result analysis (in silico) | N/A | N/A | None | LOW | Daily |

**Highest-risk step: DNA extraction from heat-inactivated specimens (minimal aerosol generation, closed, automated system).**

### PSR Classification & Modulating Factors (Steps 3–4)

Maximum PSR: LOW (all procedures — small volumes, inactivated material, closed systems, sealed tubes).

Unfavorable factors: 1 unfavorable procedural factor (daily diagnostic frequency only). Favorable factors: 6 of 9 (small volume, low concentration, minimal aerosol, low stability of inactivated material, experienced operators, adequate infrastructure).

**Only 1 unfavorable factor → escalation rule NOT triggered.**

### Risk Integration & BSL Assignment (Steps 5–6)

Severity: Moderate (inactivated RG3 material — no viable organisms present). Likelihood: Rare (Low PSR, closed systems). Risk matrix: Moderate × Rare = LOW.

Although no viable organisms are present after validated inactivation, the severity classification is conservatively maintained at Moderate rather than Low to account for the residual risk of incomplete inactivation and the high consequence severity of viable M. tuberculosis exposure. This precautionary assignment reflects the framework's principle that severity should incorporate worst-credible-case considerations for RG3-derived materials.

**BSL Assignment: BSL-2+.**

### Justification

Viable M. tuberculosis (RG3) would require BSL-3. However, PSR principles justify proportionate containment based on actual exposure risk: (1) all specimens heat-inactivated (56°C, 30 min) with validated culture-negative confirmation — no viable organisms; (2) closed automated DNA extraction (QIAcube); (3) volumes <5 mL; (4) sealed PCR tubes; (5) no cultivation/concentration of viable M. tb. BSL-2+ is supported by the proportionality principle and the WHO/BMBL precedent permitting reduced containment for inactivated RG3 materials. BSL-3 infrastructure would be disproportionate.

Inactivation was validated through culture-negative confirmation on representative sample batches, with periodic verification to ensure process reliability.

### Key Controls (Step 7, summary)

Engineering: mandatory BSC for all specimen handling; QIAcube closed system; HEPA exhaust; autoclave; self-closing restricted door. PPE: single gloves (double for specimen receipt); disposable lab coat. Administrative: SOPs including inactivation validation protocol; BSL-2 competency + molecular diagnostics certification; TB screening at hire/annually; autoclave 121°C/30 min; inactivation validation records with chain of custody; annual culture-negative spot checks. Periodic validation of inactivation (e.g., culture-based confirmation or equivalent method).

### Review Triggers (Step 8)

CRITICAL: Introduction of viable M. tb cultures requires IMMEDIATE BSL-3 upgrade. Also: extraction method change; inactivation failure (culture-positive); exposure incident; scale/sample type change; regulatory updates. Signatures: PI (Dr. M. Garcia), BSO (Dr. R. Chen), IBC (Dr. P. Lee) — Feb 2025.

# Example C — Large-Scale Recombinant AAV Production for Gene Therapy

Scenario: A gene therapy laboratory produces large-scale recombinant AAV serotype 2 (rAAV2) encoding the dystrophin gene for pre-clinical studies. Production involves triple transfection, cell lysis, clarification centrifugation, iodixanol gradient ultracentrifugation, and concentration/diafiltration. Total production volume: 500 mL per run; final titer: 10¹² particles/mL.

### Agent (Step 1)

Recombinant AAV serotype 2 (rAAV2) encoding dystrophin (therapeutic gene). Wild-type AAV2: RG1–2 (low pathogenicity, no disease association in healthy adults; opportunistic in immunocompromised). Recombinant construct: replication-incompetent, helper-dependent. Classification: RG2 (precautionary, consistent with BMBL and NIH Guidelines for rAAV used in gene therapy applications). Not a GMO under EU Directive 2009/41/EC definitions applicable to contained use for this system. Key biological traits: extremely high environmental stability (resistant to heat, detergents, pH extremes); small particle size (25 nm, remains aerosolized); no envelope (non-lytic release); no known treatment for high-dose AAV exposure.

### Procedures (Step 2) and PSR Classification & Modulating Factors (Steps 3–4)

Maximum PSR: HIGH (clarification centrifugation, iodixanol gradient ultracentrifugation, concentration/diafiltration). Unfavorable modulating factors: (1) very large volume (500 mL/run); (2) extremely high final titer (10¹² particles/mL); (3) monthly production frequency; (4) extreme environmental stability of AAV (critical factor — resistant to standard inactivation). Favorable factors: experienced operator (>2 years), dedicated production suite, sealed ultracentrifuge buckets available. ≥2 unfavorable procedural factors → escalation rule triggered. Note: extreme environmental stability is the critical factor elevating this RG2 agent beyond standard BSL-2.

### Risk Integration & BSL Assignment (Steps 5–6)

Severity: Moderate (RG2, non-replicating, no known AAV pathology in healthy adults). Likelihood: Very Likely (High PSR + 4 unfavorable factors including extreme stability). Risk matrix: Moderate × Very Likely = HIGH. BSL Assignment: BSL-2+ (some institutional protocols recommend BSL-3 for large-scale AAV production; institutional review required). Justification: Although nominally RG2, AAV’s extreme environmental persistence and 25 nm particle size create a persistent contamination risk disproportionate to its nominal classification. Large-scale production at 10¹² particles/mL with aerosol-generating steps requires enhanced containment beyond standard BSL-2. The BSL-2+ assignment includes a dedicated production room with negative pressure (recommended), mandatory Class II BSC for all open manipulations, sealed ultracentrifuge rotors, comprehensive surface decontamination with 0.5% bleach (note: many standard disinfectants are ineffective against non-enveloped AAV), and quarterly environmental monitoring by surface PCR.

### Key Controls & Review Triggers (Steps 7–8)

Engineering: dedicated BSL-2 suite (separate room); negative pressure recommended; all open steps in Class II BSC; sealed ultracentrifuge buckets mandatory; 10-min post-centrifugation wait before tube opening; HEPA exhaust; autoclave. PPE: double gloves mandatory; disposable gown; goggles or face shield; N95/FFP2 during aerosol-generating steps. Administrative: documented SOP with IBC approval; operator competency assessment; direct supervision for first 5 productions; quarterly environmental monitoring (surface PCR); pre/post operator serology for AAV; surface decontamination with 0.5% bleach (not standard ethanol). Review triggers: scale increase beyond 500 mL; titer increase above 10¹³ particles/mL; change in serotype; introduction of immunocompromised personnel; positive environmental monitoring result; annual review.

# Example D — E. coli BL21 Bacterial Pellet Sonication for Recombinant Protein

Scenario: A biochemistry laboratory produces a recombinant human protein using E. coli BL21(DE3). The protocol includes standard culture induction (500 mL), pelleting, and six cycles of probe sonication of the resuspended pellet (50 mL) for cell lysis, followed by clarification and chromatographic purification. This example illustrates the PSR principle that even RG1 agents can require enhanced containment practices for specific high-aerosol procedures.

### Agent (Step 1)

Escherichia coli BL21(DE3) expressing recombinant human protein. Classification: RG1 (non-pathogenic, well-characterized laboratory strain with reduced environmental fitness, K-12 derivative). Not a GMO for contained use classification purposes under standard biosafety frameworks when expressing non-hazardous human proteins. Key traits: non-pathogenic; low environmental persistence (laboratory-adapted); no toxin production by expressed gene product; susceptible to standard disinfectants.

### Procedures (Steps 2–4) and Risk Integration (Steps 5–6)

Culture/induction (500 mL): Low PSR. Sealed centrifugation for pellet collection: Low PSR. Pellet resuspension in lysis buffer (50 mL): Low PSR. Probe sonication (6 cycles × 30 s, ice bath, open vessel): HIGH PSR — extreme aerosol generation; bacterial concentration 10⁹–10¹⁰ CFU/mL. Clarification centrifugation: Moderate PSR. Chromatographic purification: Low PSR. Maximum PSR: HIGH (sonication step). Unfavorable modulating factors for sonication step: (1) extremely high bacterial concentration (10⁹–10¹⁰ CFU/mL); (2) open vessel sonication (no containment); (3) weekly frequency. Favorable: RG1 agent, low environmental persistence, favorable operator experience. ≥2 unfavorable factors → escalation triggered for sonication step only. Severity: Low (RG1). Likelihood: Likely (High PSR, high concentration). Risk matrix: Low × Likely = Moderate.

### BSL Assignment, Controls & Review Triggers (Steps 6–8)

BSL Assignment: BSL-1 for all standard manipulations; BSL-2 practices required exclusively for the sonication step. Justification: E. coli BL21 is RG1 and would ordinarily require only BSL-1 throughout. However, probe sonication of concentrated pellets (10⁹–10¹⁰ CFU/mL) generates massive aerosol loads. The PSR framework’s procedure-specific logic mandates enhanced containment for this step, even with a non-pathogenic agent. This is the key illustrative value of this case: the same laboratory, same organism, same experiment requires two different containment levels for different procedural steps. Implementation note: many institutions currently perform RG1 sonication under standard BSL-1 conditions. PSR-based assessment reveals this represents a preventable exposure risk. Key controls for sonication step: enclosed sonicator or Class II BSC; 10-min post-sonication wait; N95/FFP2 respiratory protection during setup/cleanup; eye protection; surface decontamination with 70% ethanol; consider cup-horn sonicator as primary risk-elimination measure. Review triggers: switch to cup-horn system (re-evaluate downward); pellet volume >100 mL; concentration >10¹¹ CFU/mL; new personnel.

# Example E — Routine HIV-1 Manipulation: Procedural Risk Stratification (Same Agent, Two BSL Outcomes)

Scenario: A virology laboratory performs both routine HIV-1 cell infection experiments (Sub-protocol A) and high-titer stock preparation by ultracentrifugation (Sub-protocol B). Both involve wild-type, replication-competent HIV-1 (RG3). This example is the most illustrative of the PSR principle: the same RG3 agent, handled in the same institutional environment, receives two different BSL assignments based exclusively on procedural exposure differences.

### Agent (Step 1)

HIV-1 (wild-type, replication-competent). RG3 (WHO, BMBL). No genetic modification. Key traits: strictly parenteral/mucosal transmission (no airborne route — critical favorable factor for PSR); moderate environmental stability at high titer; effective post-exposure prophylaxis (PEP) and antiretroviral therapy available (reduces severity); infectious dose low by parenteral route but non-zero by mucous membrane splash.

### Sub-Protocol A: Routine Cell Infection (Steps 2–6)

Procedures: thawing/maintenance of HIV-1 stocks; infection of CD4⁺ T cell lines at MOI 0.01–0.1 in 24-well plates (Class II BSC); incubation 48–72 h (sealed); supernatant harvesting for p24/TCID₅₀ (pipetting in BSC, no aerosol); inactivation with 1% Triton X-100. Total volume: 5–10 mL. Maximum PSR: Moderate. Modulating factors: 1 unfavorable (low-moderate working titer). Favorable: no airborne route; low volume (5–10 mL); all manipulations in Class II BSC; PEP available; operator >1 year experience. Escalation: NOT triggered (only 1 unfavorable factor). Severity: Moderate (RG3, no airborne). Likelihood: Possible (Moderate PSR, contained). Risk matrix: Moderate × Possible = Moderate. BSL Assignment: BSL-2+. Justification: Absence of airborne transmission, low working volumes, and complete BSC containment reduce procedural exposure probability below the threshold requiring full BSL-3 infrastructure. Consistent with BMBL (2020) and WHO (2020) guidance for routine HIV research in non-clinical settings.

### Sub-Protocol B: Virus Concentration and High-Titer Stock Preparation (Steps 2–6)

Procedures: large-volume supernatant collection (50–200 mL); ultracentrifugation for viral pellet concentration (rotor unsealing generates aerosol); resuspension and aliquoting at 10⁷–10⁸ TCID₅₀/mL; titration assays. Maximum PSR: HIGH (ultracentrifugation + high-titer handling). Modulating factors: 3 unfavorable (large volume 50–200 mL; very high titer 10⁷–10⁸ TCID₅₀/mL; aerosol risk from rotor unsealing). ≥2 unfavorable factors → escalation triggered. Severity: Moderate (RG3, no airborne). Likelihood: Very Likely (High PSR + 3 unfavorable factors). Risk matrix: Moderate × Very Likely = HIGH. BSL Assignment: BSL-3. Justification: Aerosol-generating ultracentrifugation, very high viral titers, and large volumes elevate procedural exposure probability to a level requiring full BSL-3: directional airflow, HEPA-filtered exhaust, sealed centrifuge rotors, and respiratory protection. Same RG3 agent as Sub-protocol A; different BSL assignment determined exclusively by procedural exposure differences. This is the definitive illustration of PSR’s rejection of automatic RG→BSL correlation.

### Key Controls & Review Triggers (Steps 7–8)

Sub-protocol A (BSL-2+): all manipulations in Class II BSC; double gloves + eye/face protection; sharps safety (no needles/Pasteur pipettes); immediate spill decontamination (10% bleach, 30 min); liquid waste bleach-treated before disposal; 24-hour PEP access; incident reporting system; documented operator training in HIV transmission and PEP. Sub-protocol B (BSL-3 additional): full BSL-3 facility with directional airflow and HEPA exhaust; sealed centrifuge rotors or safety cups mandatory; FFP2/N95 during rotor handling; all high-titer work in Class II BSC with additional respiratory protection; decontamination of all centrifuge components after each run; restricted access during concentration; secondary containment for all high-titer aliquots. Review triggers (both): change in virus strain; MOI increase above 1; volume above 250 mL; new personnel; incident/exposure; regulatory updates; annual review.

# Example F — CRISPR-Cas9 Gene Editing: Lentiviral Delivery vs. RNP Electroporation

Scenario: A genome engineering laboratory implements CRISPR-Cas9 editing of human cell lines using two alternative delivery modalities: (A) a third-generation lentiviral vector encoding Cas9 and guide RNA, and (B) pre-assembled ribonucleoprotein (RNP) complexes delivered by electroporation. Both achieve equivalent genome-editing outcomes. This example illustrates that biosafety requirements are driven by procedural risk profile, not by the sophistication or category of the biotechnology used.

### Sub-Protocol F-A: Lentiviral Delivery (Steps 1–6)

Agent: Third-generation lentiviral vector encoding Cas9/gRNA (HIV-1 backbone, replication-defective). Classification: RG2 (documented attenuation; same classification logic as Example A). Procedures: HEK293T transfection for vector production (Moderate PSR); viral supernatant harvest (Moderate PSR); ultracentrifugation or concentration (HIGH PSR); transduction of target cells in Class II BSC (Moderate PSR). Maximum PSR: HIGH. Modulating factors: 2–3 unfavorable (high viral titers 10⁷–10⁹ TU/mL; aerosol generation during concentration; repeated production cycles). ≥2 unfavorable → escalation triggered. Severity: Moderate (RG2, replication-defective). Likelihood: Very Likely (High PSR + titers). Risk matrix: Moderate × Very Likely = HIGH. BSL Assignment: BSL-2+. Controls: mandatory Class II BSC; sealed centrifuge rotors/cups; double gloves; eye protection; controlled access during production; documented competency in lentiviral vector biosafety.

### Sub-Protocol F-B: RNP Electroporation (Steps 1–6)

Agent: Pre-assembled Cas9–gRNA ribonucleoprotein complexes. Classification: RG1 (non-replicative protein–RNA complex; no infectious capability; no viral components; rapid intracellular degradation). Procedures: preparation of Cas9–gRNA complexes (Low PSR); electroporation using closed cuvette systems (Low–Moderate PSR); post-electroporation cell recovery (Low PSR). Maximum PSR: Low. Modulating factors: no unfavorable factors (small volume <5 mL; non-replicative; closed electroporation systems; rapid RNP degradation; no environmental persistence). Escalation: NOT triggered. Severity: Low (RG1). Likelihood: Rare (Low PSR, closed systems, transient activity). Risk matrix: Low × Rare = Negligible. BSL Assignment: BSL-1 (enhanced practices recommended for electroporation safety). Justification: Elimination of replication-capable intermediates and minimal environmental persistence markedly reduce procedural exposure probability. Standard BSL-1 microbiological practices suffice with attention to electroporation-specific hazards (electrical safety, cuvette handling). Controls: standard BSL-1; eye protection during electroporation; routine surface decontamination; training in sharps and electroporation safety.

Comparative note (Example F): Both sub-protocols achieve equivalent CRISPR-Cas9 genome editing. Lentiviral delivery requires BSL-2+ due to viral vector production risks; RNP electroporation requires only BSL-1 due to the absence of replication-capable intermediates and minimal environmental persistence. Biosafety requirements track procedural risk, not biotechnology category. Review triggers (F-A): change in packaging system; new serotype/pseudotype; scale increase; new personnel. Review triggers (F-B): switch to viral delivery system (re-evaluate upward); introduction of pathogenic cargo genes; change in cell type.

## Comparative Summary

| **Case** | **Agent / RG** | **Max PSR** | **Unfavorable factors** | **Risk matrix** | **BSL assigned** | **PSR principle demonstrated** |
| --- | --- | --- | --- | --- | --- | --- |
| A | HIV-1 lentiviral vector (3rd gen, replication-defective) / RG2 | HIGH (ultracentrifugation, high titer) | 4 → escalation triggered | Moderate × Very Likely = HIGH | BSL-2+ (escalation from BSL-2) | Procedure-driven escalation despite low RG |
| B | M. tuberculosis / RG3 (heat-inactivated, no viable organisms) | LOW (all procedures closed, small volume) | 1 → escalation NOT triggered | Moderate × Rare = LOW | BSL-2+ (reduction from BSL-3) | Proportionality-driven reduction despite high RG |
| C | Recombinant AAV2 (dystrophin) / RG2 | HIGH (ultracentrifugation, 500 mL, 10¹² particles/mL) | 4 → escalation triggered | Moderate × Very Likely = HIGH | BSL-2+ | Scale + extreme environmental persistence override nominal RG2 |
| D | E. coli BL21(DE3) / RG1 | HIGH (sonication step only); LOW for all other steps | 2–3 (sonication step only) → triggered | Low × Likely = Moderate | BSL-1 (standard) / BSL-2 practices (sonication step only) | Step-specific escalation: RG1 agent requires enhanced practices for extreme-aerosol procedure only |
| E-A | HIV-1 wild-type / RG3 — routine cell infection (5–10 mL, BSC, no aerosol-generating steps) | MODERATE | 1 → NOT triggered | Moderate × Possible = Moderate | BSL-2+ | No airborne route + low volume + BSC containment reduce RG3 below BSL-3 threshold |
| E-B | HIV-1 wild-type / RG3 — high-titer concentration (same agent as E-A) | HIGH (ultracentrifugation, 50–200 mL, 10⁷–10⁸ TCID₅₀/mL) | 3 → escalation triggered | Moderate × Very Likely = HIGH | BSL-3 | Same RG3 agent as E-A; aerosol + high titer mandate full BSL-3 — definitive PSR demonstration |
| F-A | CRISPR-Cas9 lentiviral delivery / RG2 (replication-defective HIV-1 vector) | HIGH (vector production + ultracentrifugation) | 2–3 → escalation triggered | Moderate × Very Likely = HIGH | BSL-2+ | Viral vector production risk; replication-defective status preserves BSL-2+ vs BSL-3 |
| F-B | CRISPR-Cas9 RNP electroporation / RG1 (non-replicative protein–RNA complex) | LOW (<5 mL, non-replicative, closed electroporation systems) | 0 → NOT triggered | Low × Rare = Negligible | BSL-1 | Same editing outcome as F-A; BSL-1 reflects elimination of all replicative intermediates |

*Supplementary Appendix S2. Six worked examples demonstrating PSR framework applications across RG1–3, Low–High procedural risk, and diverse biological systems: (A) containment escalation for a low-hazard agent driven by high-risk procedures; (B) proportionate containment reduction for a high-hazard agent handled through validated low-risk procedures; (C) scale-driven escalation for an environmentally persistent RG2 vector; (D) procedure-specific escalation for a non-pathogenic agent (RG1 sonication); (E) same-agent dual BSL assignment illustrating the core PSR principle; and (F) technology-neutral comparison of two CRISPR delivery modalities with divergent risk profiles.*

**Supplementary Appendix S3**

**Interactive PSR Assessment Platform**

An interactive web-based tool has been developed to support the implementation of the standardized PSR framework. The platform is freely accessible at:

<https://rafael-67.github.io/psr-tool/>

*The platform is a decision-support instrument and does not replace formal institutional risk assessment procedures.*

## Architecture

| **Module** | **Function** | **Users** |
| --- | --- | --- |
| **A — Standalone Tool** | Individual PSR evaluations, bilingual EN/ES, offline-capable, PDF/TXT export | PIs, biosafety officers, lab personnel |
| **B — Multi-User Platform** | Persistent storage*, institutional profiles, audit trail, assessment lifecycle (draft → finalized → shared) | IBCs, multi-lab facilities |
| **C — Data Analytics** | BSL distributions, escalation drivers, Cohen's κ for inter-rater reliability, confusion matrices, temporal trends, CSV export | Researchers, regulatory bodies |
| *Persistence is browser-dependent; see Limitations. | | |

## Framework Implementation

The platform encodes Tables 6–8 from the main article, including the PSR classification matrix, the ≥2 unfavorable factors escalation rule, the RG3* designation for replication-defective GMOs derived from RG3 agents, and the Agent–PSR combination matrix for BSL assignment. Pre-loaded worked examples from Supplementary Appendix S2 are available for training.

## Inter-Rater Reliability Module

Module C computes Cohen's κ from paired BSL assignments by independent evaluators assessing identical agent–procedure scenarios. The module reports observed agreement (P₀), expected agreement by chance (Pₑ), and κ = (P₀ − Pₑ) / (1 − Pₑ), with interpretation benchmarks per Landis and Koch (1977): κ < 0.20 poor; 0.21–0.40 fair; 0.41–0.60 moderate; 0.61–0.80 substantial; 0.81–1.00 almost perfect. This supports the multi-institutional validation protocol described in the Methods section (Validation approach) and discussed in Section 6.3 of the main article.

## Technical Specifications

Client-side web application (HTML5/CSS3/JavaScript). Hosted on GitHub Pages (static, no server-side processing). No external data transmission. Session data is held in memory during use; completed assessments must be exported (PDF/TXT) at the end of each session to be retained, as no automatic persistence is implemented in the current version. Compatible with Chrome, Firefox, Safari, Edge (last 2 major versions). Bilingual EN/ES. Offline-capable after initial load. Open source (MIT license).

## Limitations

BSL recommendations require formal IBC approval. The Agent RG classification must be determined by the user using authoritative sources. The current version does not implement automatic data persistence: assessment data exists only in active session memory and is lost when the browser tab is closed or the page is refreshed. Users must export assessment records (PDF/TXT) at the end of each session; export after every completed evaluation is mandatory for audit trail integrity in institutional use. The tool operates entirely client-side with no external data transmission or server-side storage. For environments requiring guaranteed data persistence, multi-device access, and institutional audit trail management, deployment on a server-based infrastructure with a database backend is recommended as a future development priority.

The platform has not undergone formal software validation (IEC 62304) and is provided as a research and educational tool. The platform is a decision-support architecture and **does not replace the professional judgment** of the Institutional Biosafety Officer (BSO) or the specific requirements of local regulatory authorities.

*Reference: Landis JR, Koch GG. The measurement of observer agreement for categorical data. Biometrics. 1977;33(1):159–174.*

*Supplementary Appendix S3. Technical description of the interactive PSR Assessment Platform implementing the 8-step Procedure-Specific Risk framework as a guided web-based workflow with integrated data analytics and inter-rater reliability computation. Freely accessible at:* [*https://rafael-67.github.io/psr-tool/*](https://rafael-67.github.io/psr-tool/)

**Supplementary Appendix S4**

**Table S1. Five Fundamental Principles of PSR: International Cross-Source Alignment. Explicit endorsement: principle stated and operationalized with specific criteria in the source. Implicit endorsement: principle consistent with the source framework but not formally articulated as a distinct requirement. Classification based on procedure focus ratings (Table 2) and terminological evidence (Table 4) in the main article.**

| **Principle** | **Core Tenet** | **Explicit endorsement** | **Implicit endorsement** |
| --- | --- | --- | --- |
| **1. Multifactorial Integration** | Risk = f(Agent, Procedure, Context) | WHO 2020; BMBL 2020; CDC 2024; INSST Guide 2024; NTP 1201; NTP 1202; AAMI 2016; Select Agents 2025 | RD 664/1997; NTP 875 |
| **2. No RG-BSL Automaticity** | RG classification ≠ automatic BSL assignment | BMBL 2020; CDC 2024; INSST Guide 2024; NTP 1201; AAMI 2016; Select Agents 2025 | WHO 2020; RD 664/1997; NTP 875; NTP 1202 |
| **3. Dynamic Revisability** | Continuous review upon changes | RD 664/1997; BMBL 2020; WHO 2020; INSST Guide 2024; Select Agents 2025; NTP 1201 | CDC 2024; NTP 875; NTP 1202; AAMI 2016 |
| **4. Mandatory Documentation** | Written justification required | RD 664/1997; INSST Guide 2024; BMBL 2020; Select Agents 2025; NTP 1201 | WHO 2020; CDC 2024; NTP 875; NTP 1202; AAMI 2016 |
| **5. Proportionate Containment** | Controls match actual operational risk | WHO 2020; BMBL 2020; CDC 2024; INSST Guide 2024; NTP 1201; Directive 2000/54/EC | RD 664/1997; NTP 875; NTP 1202; AAMI 2016; Select Agents 2025 |
